# LAMP2A regulates the loading of proteins into exosomes

**DOI:** 10.1126/sciadv.abm1140

**Published:** 2022-03-25

**Authors:** João Vasco Ferreira, Ana da Rosa Soares, José Ramalho, Catarina Máximo Carvalho, Maria Helena Cardoso, Petra Pintado, Ana Sofia Carvalho, Hans Christian Beck, Rune Matthiesen, Mónica Zuzarte, Henrique Girão, Guillaume van Niel, Paulo Pereira

**Affiliations:** 1 Proteostasis and Proteolytic Signalling Lab, Chronic Diseases Research Centre (CEDOC), NOVA Medical School, Faculdade de Ciencias Medicas, Universidade NOVA de Lisboa, Lisbon, Portugal.; 2 Fish Facility, CEDOC, NOVA Medical School, Faculdade de Ciencias Medicas, Universidade NOVA de Lisboa, Lisbon, Portugal.; 3 Computational and Experimental Biology Group, CEDOC, NOVA Medical School, Faculdade de Ciencias Medicas, Universidade NOVA de Lisboa, Lisbon, Portugal.; 4 Centre for Clinical Proteomics, Department of Clinical Biochemistry and Pharmacology, Odense University Hospital, Odense, Denmark.; 5 University of Coimbra, Coimbra Institute for Clinical and Biomedical Research (iCBR), Faculty of Medicine, Center for Innovative Biomedicine and Biotechnology (CIBB), Clinical Academic Centre of Coimbra (CACC), Coimbra, Portugal.; 6 Université de Paris, Institute of Psychiatry and Neuroscience of Paris (IPNP), INSERM U1266, F-75014 Paris, France.; 7 GHU Paris Psychiatrie et Neurosciences, Hôpital Sainte Anne, F-75014 Paris, France.

## Abstract

Exosomes are extracellular vesicles of endosomal origin that are released by practically all cell types across metazoans. Exosomes are active vehicles of intercellular communication and can transfer lipids, RNAs, and proteins between different cells, tissues, or organs. Here, we describe a mechanism whereby proteins containing a KFERQ motif pentapeptide are loaded into a subpopulation of exosomes in a process that is dependent on the membrane protein LAMP2A. Moreover, we demonstrate that this mechanism is independent of the ESCRT machinery but dependent on HSC70, CD63, Alix, Syntenin-1, Rab31, and ceramides. We show that the master regulator of hypoxia HIF1A is loaded into exosomes by this mechanism to transport hypoxia signaling to normoxic cells. In addition, by tagging fluorescent proteins with KFERQ-like sequences, we were able to follow the interorgan transfer of exosomes. Our findings open new avenues for exosome engineering by allowing the loading of bioactive proteins by tagging them with KFERQ-like motifs.

## Teaser

LAMP2A targets cytosolic proteins to extracellular vesicles to modulate interorgan communication.

## INTRODUCTION

Exosomes are nanosized vesicles of 40 to 160 nm in diameter that are secreted by most cell types to the extracellular space (*1*). Exosomes containing lipids, metabolites, proteins, and RNA can travel and transfer cellular information from one cell to another (*1*–*3*) in diverse biological processes such as immune function, viral infection, metabolic regulation, tumor metastasis, and neurodegeneration (*4*). In addition, circulating exosomes have several potential uses, such as disease biomarkers in liquid biopsies, and efforts are being made to create engineered exosomes for a variety of therapeutic approaches such as vaccines and gene therapy or to efficiently target undruggable proteins.

Exosomes are formed by the inward budding of the endosomal limiting membrane to create vesicle-laden endosomes containing intraluminal vesicles (ILVs), referred to as multivesicular bodies (MVBs) (*5*). Fusion of a subset of these MVBs with the plasma membrane leads to the release of ILVs to the extracellular space as exosomes (*5*). After secretion from the originating cell, exosomes can dock and fuse with, or be internalized by, other cells to deliver their cargo (*5*, *6*).

ILV biogenesis was considered to be largely mediated by the ESCRT (endosomal sorting complex required for transport) machinery (*1*, *5*, *7*). However, ESCRT-depleted cells can still generate ILVs (*8*). Alternative mechanisms have been reported to assist in ILV formation and cargo sorting, such as the sphingolipid ceramide (*8*), the tetraspanin CD63, the Toll-like receptor trafficking chaperone UNC93B1, or the syndecan-syntenin-Alix pathway (*1*, *9*–*12*). Notably, there is ample evidence suggesting that the cargo repertoire of exosomes does not necessarily reflect the cytosolic contents of the originating cell (*13*, *14*), indicating the existence of an active soluble cargo selection or triage mechanism. However, the mechanisms controlling selection and sorting of soluble cytoplasmic components into exosomes remain largely unknown.

In this study, we show that proteins containing amino acid sequences biochemically related to the KFERQ motif (*15*) are loaded into a subpopulation of exosomes. We identified the following as essential components of the machinery: the lysosome-associated membrane protein 2, isoform A (LAMP2A), previously recognized as a protein that targets KFERQ-containing substrates to the lysosome (*15*), and the molecular chaperone HSC70 (heat shock 70-kDa protein 8). In addition, we demonstrate that the loading of proteins takes place at the early endosomal membrane and relies on the endosomal proteins CD63, Alix, Syntenin-1, and RAB31 (Ras-related protein 31) rather than on components of the ESCRT machinery proteins such as TSG101 (tumor susceptibility gene 101 protein) and VPS4b (Vacuolar protein sorting-associated protein 4B). We further show that this mechanism is of biological relevance in animals. For example, we showed that the hypoxia master regulator HIF1A (hypoxia-inducible transcription factor 1 α) is loaded into exosomes by the action of its KFERQ-like motif and the presence of LAMP2A in endosomes, to transfer HIF1A transcriptional activity from hypoxic to normoxic cells, both in vitro and in zebrafish. Moreover, our findings enabled us to develop tools to track the transfer of exosomes from one tissue to another in zebrafish larvae, opening new possibilities for the study of exosome-mediated interorgan communication.

## RESULTS

### LAMP2A is required for the sorting of selected proteins into extracellular vesicles

**Fig. 1.**
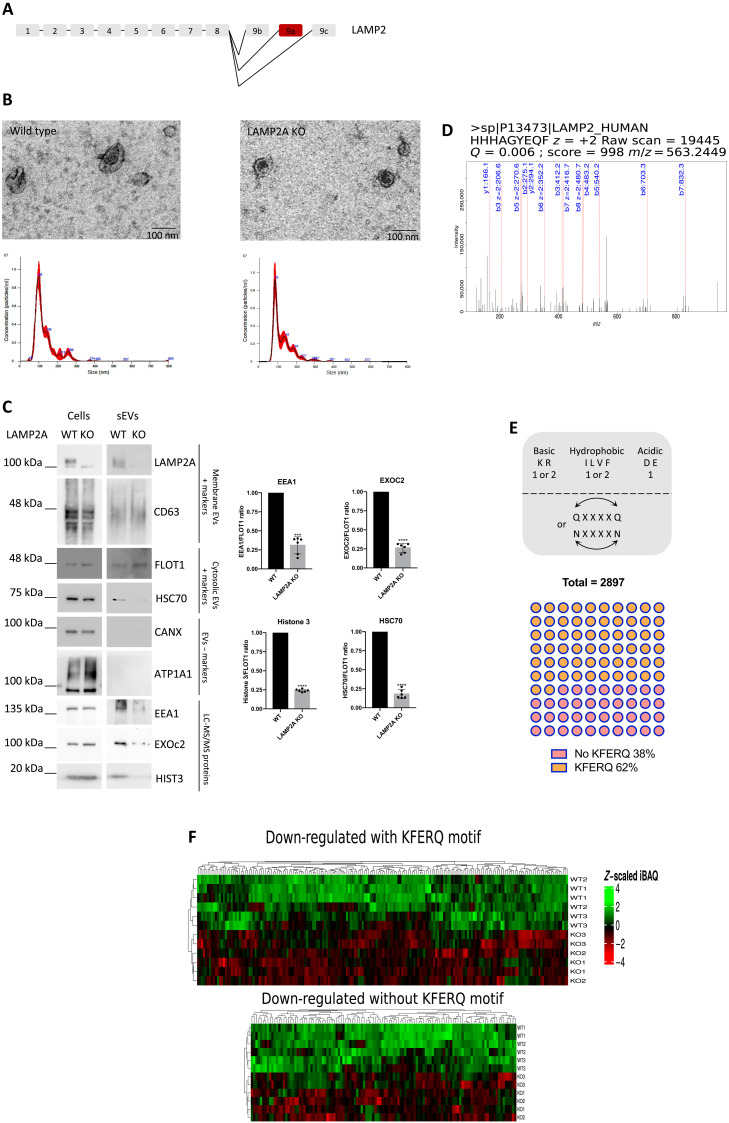
LAMP2A KO leads to the down-regulation of KFERQ-containing proteins in sEVs. (**A**) Schematic representation of LAMP2 genomic sequence. There are three isoforms of LAMP2 (A, B, and C) originated by the alternative splicing of exon 9. In red is the exon for the LAMP2A isoform. ARPE-19 cells were cultured in exosome-depleted medium. sEVs were isolated from cell culture supernatants. (**B**) TEM images show isolated exosomes. Graphs show particle number and size using nanoparticle tracking system (NanoSight). LAMP2A KO maintains particle size and number. (**C**) WB of cell extracts and sEV fractions blotted with antibodies raised against membrane EV-positive markers (CD63 and LAMP2A), cytosolic EV-positive markers (HSC70 and FLOT1), EV-negative markers [CANX (calnexin) and ATP1A1 (ATPase Na+/K+ Transporting Subunit Alpha 1)], and KFERQ-containing proteins. LAMP2A KO leads to a decrease in the protein levels of EEA1, EXOc2, and histone 3, which contain at least one KFERQ motif each, as well as HSC70. (**D**) Raw MS/MS fragmentation spectrum of the peptide HHHAGYEQF, exclusively from LAMP2 isoform A, present only in WT samples. (**E**) Schematic representation shows the rules for the identification of putative KFERQ motifs. Graph shows the percentage of proteins with at least one KFERQ motif in their sequence that is present in sEVs. (**F**) Heatmaps of down-regulated proteins in sEVs after LAMP2A KO. Down-regulated proteins (67%) contain KFERQ motifs. All samples were analyzed under the same experimental conditions. The results represent means ± SD of at least *n* = 3 independent experiments (***P < 0.001 and ****P < 0.0001).

To investigate the impact of LAMP2A in exosome protein cargo, we used the CRISPR-Cas9 system to knock out (KO) the LAMP2A gene in a human cell line with normal chromosome number, ARPE-19. LAMP2A originates from the alternative splicing of the LAMP2 gene, which contains nine exons, including three different splice variants of the exon 9 (A, B, and C) (Fig. 1A). All splice variants share a common luminal domain but contain distinct cytosolic and transmembrane regions. We were able to isolate several clones of ARPE-19 cells without LAMP2A while maintaining the expression of other LAMP2 isoforms (fig. S1A). We used clone 8 as wild type (WT) and clone 28 as LAMP2A KO (fig. S1A).

Extracellular vesicles (EVs) from the media supernatants of WT and LAMP2A KO ARPE-19 cells were isolated for mass spectrometry (MS)–based proteome analysis. Note that there is still a lack of consensus on specific markers for exosomes, and the most common method used for exosome isolation, ultracentrifugation, is unable to separate exosomes from other populations of small EVs such as microvesicles. Following EV guidelines (*16*), we were able to isolate vesicles smaller than 200 nm (including exosomes), henceforth referred to as sEVs. We confirmed that the size of the secreted sEVs of WT and LAMP2A KO cells is within the range of exosomes (~100 nm) by transmission electron microscopy (TEM) and by laser scattering using NanoSight tracking system (Fig. 1B). TEM of ARPE-19 cells further showed that MVB morphology is maintained after LAMP2A KO (fig. S1B), while the rate of fusion between late endosomes (LEs)/MVBs and lysosomes is unaffected by LAMP2A KO, as measured by the golden standard procedure to follow endosome fusion with lysosomes using fluorescent dextran of a specific size (10,000 Da; fig. S1C) (*17*).

On the other hand, Western blotting (WB) of 2.5 μg of whole-cell lysates and 100% of isolated sEVs secreted by 40.0 × 10^6^ cells confirmed the presence of membrane proteins of endosomal origin, CD63 and LAMP2A, and the cytosolic proteins associated to EVs Flotillin-1 (FLOT1) and HSC70, as well as the absence of the endoplasmic reticulum marker calnexin and the plasma membrane marker Na^+^- and K^+^-dependent adenosine triphosphatase (Na^+^,K^+^-ATPase; Fig. 1C) (*16*, *18*). Liquid chromatography–tandem MS (LC-MS/MS) of isolated sEVs confirmed the presence of the peptide fragment HHHAGYEQF, specifically corresponding to LAMP2A, only in WT sEVs (Fig. 1D).

With the LC-MS/MS analysis of sEV fractions, we identified 2897 proteins (table S1). Accordingly, the identified proteins were enriched in EV markers to the same level as in previously published EV studies (fig. S1D) (*19*–*21*). Because LAMP2A is reported to bind to proteins that contain KFERQ-like motifs (*15*), we next searched the identified proteins for these amino acid sequences. KFERQ motifs are degenerated pentapeptide sequences recognized by the HSC70 (Fig. 1E). Initial estimates of the abundance of KFERQ motifs in the proteome indicated that ~25% of proteins contain such active/exposed motifs (*22*). In our isolated sEVs, approximately 62% of all proteins contained a putative KFERQ motif (Fig. 1E and table S2), representing a 2.5-fold enrichment. Further analysis of sEVs secreted from WT and LAMP2A KO cells showed that 303 proteins were significantly down-regulated (*P* < 0.05) in LAMP2A KO sEVs and that 203 of the down-regulated proteins (67%) contained a putative KFERQ motif (Fig. 1F and table S3). WB of LAMP2A KO sEVs showed a reduction in HSC70 levels, while confirming a decrease in the levels of randomly selected proteins from the LC-MS/MS data (Fig. 1C). These findings suggest that LAMP2A KO leads to a decrease in the levels of proteins containing KFERQ-like motifs in sEVs.

### Loading of KFERQ-like motif–tagged proteins in EVs is dependent on LAMP2A and HSC70

**Fig. 2.**
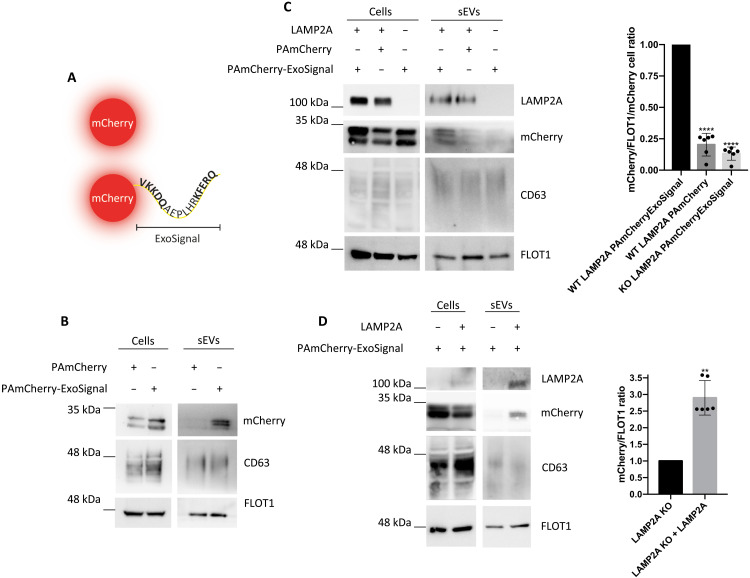
mCherry presence in sEVs depends on both the ExoSignal and LAMP2A. (**A**) Schematic representation of mCherry tagged with the ExoSignal sequence. WT and LAMP2A KO cells were transduced with lentiviral particles for the expression of either mCherry or mCherry-ExoSignal. sEVs were isolated from cell culture supernatants. (**B** and **C**) WB of cell extracts and isolated sEVs blotted with antibodies raised against CD63, FLOT1, LAMP2A, and mCherry. The addition of the ExoSignal increases the levels of mCherry in sEVs. (C) KO of LAMP2A decreases the levels of mCherry-ExoSignal into exosomes. (**D**) ARPE-19 cells, KO for LAMP2A, were transduced with lentiviral particles to express human LAMP2A. Results show that rescuing LAMP2A expression is sufficient to rescue the presence of mCherry-ExoSignal in sEVs. All samples were analyzed under the same experimental conditions. The results represent means ± SD of at least *n* = 3 independent experiments (**P < 0.01 and ****P < 0.0001).

To address the mechanisms involved in the targeting of proteins containing KFERQ motifs to EVs, we used chimeric proteins consisting of a fluorescent protein, such as mCherry, and the KFERQ motifs of α-synuclein (VKKDQ) and ribonuclease A (KFERQ) separated by a peptide spacer (Fig. 2A), based on previous reports (*23*, *24*). WB of sEV fractions from cells expressing either mCherry alone or mCherry fused to the targeting peptide, hereafter referred to as ExoSignal, showed that the ExoSignal tag increases mCherry presence in sEVs by about sevenfold (Fig. 2, B and C). On the other hand, KO of LAMP2A from cells expressing mCherry-ExoSignal prevented the loading of the chimeric protein into sEVs (Fig. 2C). In addition, the rescue of LAMP2A expression was sufficient to restore the levels of mCherry-ExoSignal in sEVs (Fig. 2D). mCherry-ExoSignal was present in sEV fractions positive for CD63 and with typical exosomal densities (fig. S2A). Moreover, incubation of sEVs with trypsin showed that mCherry is resistant to degradation, while the sEV membrane protein Cx43 (*6*) was not, suggesting that mCherry-ExoSignal is incorporated into the lumen of sEVs (fig. S2B). To further confirm that mCherry-ExoSignal is loaded into EVs, we down-regulated RAB27 to inhibit the release of EVs (*18*, *25*). Rab27 depletion decreased sEV secretion and the levels of mCherry-ExoSignal present in the sEV fractions (fig. S2C).

**Fig. 3.**
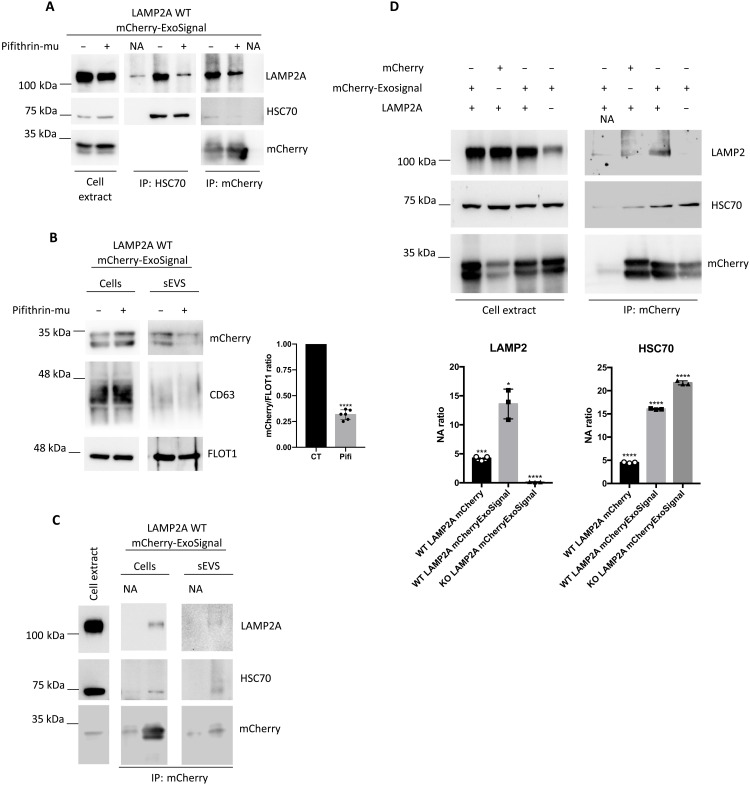
HSC70 is necessary for the presence of mCherry-ExoSignal in sEVs. WT and LAMP2A KO ARPE-19 cells were transduced using lentiviral particles containing vectors for the expression of either mCherry or mCherry-ExoSignal. (**A**) Cells were maintained in the presence or absence or 7.5 μM Pifithrin-mu (Pifi) for 12 hours. Immunoprecipitation experiments using antibodies raised against HSC70 and mCherry show that Pifi inhibits the interaction of HSC70 with PAmCherry-ExoSignal while also decreasing the interaction with LAMP2A. (**B**) Cells were maintained in the presence or absence of 7.5 μM Pifithrin-mu for 12 hours in exosome-depleted medium. WB of exosomal fractions using antibodies raised against CD63, FLOT1, and mCherry shows that Pifi induces a decrease in the levels of mCherry-ExoSignal in sEVs. (**C**) Cells were maintained in the exosome-depleted medium for 48 hours, and sEVs were isolated from the medium supernatant. Immunoprecipitation using antibodies raised against mCherry in cell extracts and sEV samples show that PAmCherry-ExoSignal interacts with HSC70 and LAMP2A. (**D**) Immunoprecipitation in cell extracts using antibodies raised against mCherry shows that mCherry-ExoSignal, but not mCherry alone, coprecipitates with HSC70 and LAMP2A. All samples were analyzed under the same experimental conditions. The results represent means ± SD of at least *n* = 3 independent experiments (NA, no antibody; *P < 0.05, ***P < 0.001, and ****P < 0.0001).

We hypothesized that HSC70 is involved in the targeting of proteins into nascent EVs by binding and delivering proteins containing KFERQ-like motifs to endosomes. To assess whether HSC70 has a role in the loading of proteins into sEVs, we used Pifithrin-μ (Pifi), a compound that acts by inhibiting the interaction of HSC70 with its substrates (*26*). Immunoprecipitation data showed that incubation of cells with 7.5 μM Pifi led to an inhibition of the interaction between HSC70 and both mCherry-ExoSignal and LAMP2A (Fig. 3A) and a decrease in mCherry-ExoSignal protein levels in isolated sEVs (Fig. 3B). By immunoprecipitating mCherry from cells or sEVs, we confirmed that, when tagged with the ExoSignal, both HSC70 and LAMP2A coprecipitated with mCherry (Fig. 3, C and D). Overall, these data suggest that cytosolic proteins containing KFERQ motifs are loaded into EVs in a mechanism that involves both LAMP2A and HSC70.

### Proteins tagged with KFERQ-like motifs are loaded into endosomes early in the endocytic pathway

**Fig. 4.**
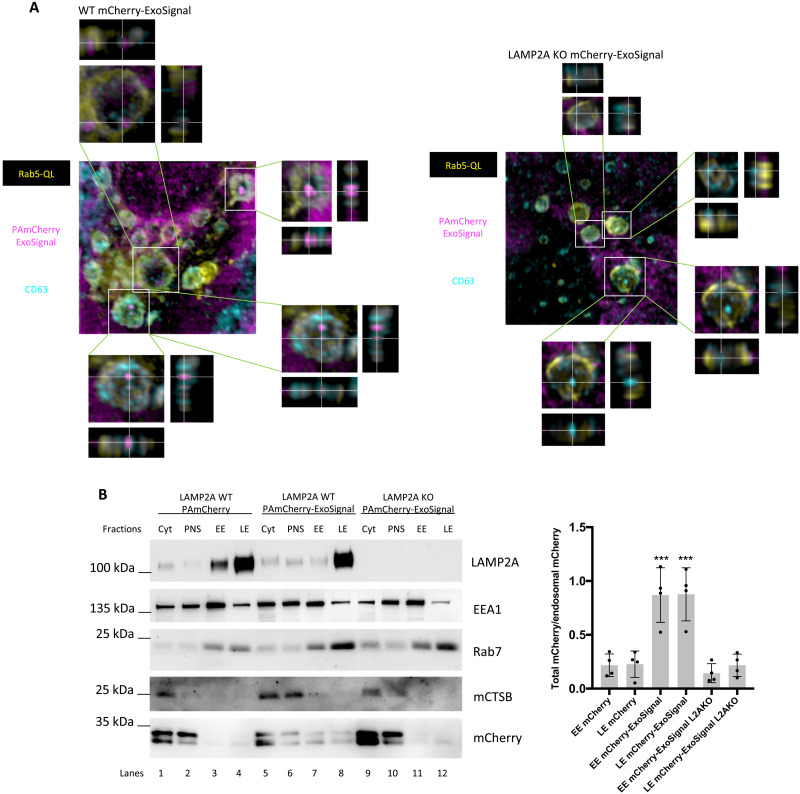
The PAmCherry-ExoSignal chimeric in ILVs. WT and KO LAMP2A ARPE-19 cells were transduced with lentiviral particles containing vectors for the expression of mCherry-ExoSignal. (**A**) Cells were transfected with Rab5QL-GFP and incubated for 30 min with antibody against CD63 extracellular loop for 30 min before fixation. Immunofluorescence using confocal microscopy shows that mCherry-ExoSignal puncta localizes in Rab5QL-GFP compartments, inside CD63-positive ILVs when LAMP2A is present. 3D images were reconstructed using Imaris software. (**B**) Sucrose discontinuous gradients were performed as described in the protocol. Cytoplasmic (Cyt), postnuclear supernatant (PNS), and EE- and LE-enriched fractions of WT and KO LAMP2A cells were loaded into an SDS-PAGE. WBs of isolated fractions were blotted with antibodies raised against the EE marker EEA1, the LE marker Rab7, the lysosome marker cathepsin B (CTSB; mature form), LAMP2A, and mCherry. (A) PAmCherry-ExoSignal, but not PAmCherry, is present in both EE and LE compartments (compare lanes 3 and 4 with lanes 7 and 8), only when LAMP2A is present (compare lanes 7 and 8 with 11 and 12). The results represent means ± SD of at least *n* = 3 independent experiments (***P < 0.001).

While some EVs such as microvesicles originate from shedding of the plasma membrane, exosomes are formed by the inward invagination of the endosomal limiting membrane followed by fusion of the endosome with the plasma membrane. To investigate whether ExoSignal-tagged proteins are present in endosomes, we used the constitutively active mutant of Rab5 (Q79L) that blocks the conversion of early endosomes (EEs) into LEs, resulting in the formation of very large hybrid endosomes that accumulate large ILVs (*27*). Cells were incubated with anti-CD63 antibody for 30 min before fixation to exclusively label plasma membrane CD63 that becomes internalized through the endocytic pathway (*28*). Results showed that mCherry-ExoSignal localizes to Rab5QL-GFP (green fluorescent protein) compartments inside ILVs decorated with CD63 (Fig. 4A). LAMP2A KO or the absence of the ExoSignal inhibited the targeting of the PAmCherry into CD63-positive ILVs (Fig. 4A and fig. S3), while the rescue of LAMP2A expression was sufficient to restore the presence of PAmCherry-ExoSignal to ILVs (fig. S3).

Subsequently, we isolated endosomal compartments using a discontinuous sucrose gradient, according to protocol (*29*). We obtained two fractions, one enriched in the EE marker EEA1 and another enriched in the LE Rab7 (Fig. 4B). The mature form of cathepsin B (a lysosomal marker) was absent from both endosomal fractions. The data demonstrated that mCherry-ExoSignal, but not untagged mCherry, was present in both early and late endosomal compartments, only when LAMP2A was present (Fig. 4B).

**Fig. 5.**
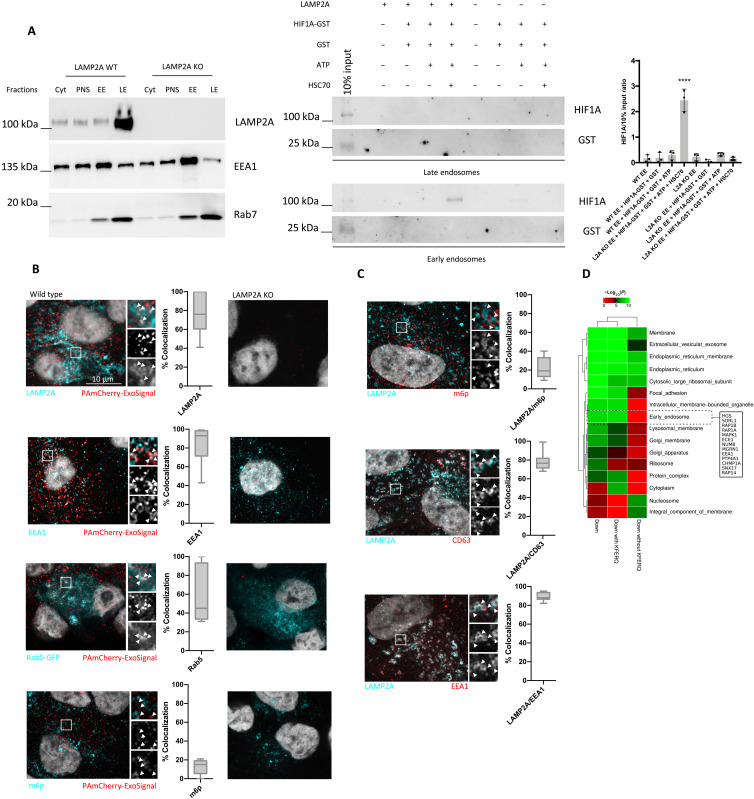
KFERQ-containing proteins are loaded into endosomes. Sucrose discontinuous gradients were performed as described in the protocol. Cytoplasmic (Cyt), PNS, and EE- and LE-enriched fractions of WT and KO LAMP2A cells were loaded into an SDS-PAGE. WBs of isolated fractions were blotted with antibodies raised against the EE marker EEA1, the LE marker Rab7, and LAMP2A. (**A**) Left: EEs and LEs without trypsin incubation. Right: HIF1A-GST was incubated with freshly isolated vesicles in the presence/absence of ATP, the molecular chaperone HSC70, and LAMP2A. Samples were treated with trypsin to degrade all the protein that was not protected by the endosomal membrane. WB using antibodies raised against the GST-tag shows that HIF1A-GST is translocated into EEs, in a LAMP2A- and HSC70-dependent manner. (**B**) Immunofluorescence using LSM 980 Airyscan confocal microscopy of cells fixed in methanol with antibodies against mCherry, LAMP2A, EEA1, and m6p or expressing Rab5-GFP. PAmCherry-ExoSignal shows high colocalization coefficient with LAMP2A, EEA1, and RAB5-GFP. KO LAMP2A cells expressing PAmCherry-ExoSignal show residual or no puncta. (**C**) Immunofluorescence using LSM 980 Airyscan confocal microscopy with antibodies against LAMP2A and either m6p, CD63, or EEA1. LAMP2A colocalizes preferentially with CD63 and EEA1. (**D**) Functional enrichment analysis matching protein subcellular compartment localization, as defined in gene ontology, against proteins down-regulated in EVs upon LAMP2A KO. EE proteins are among the most down-regulated. Furthermore, all EE down-regulated proteins contained the KFERQ motif. The results represent means ± SD of *n* = 3 independent experiments (****P < 0.0001).

Next, we used the endosomal fractions for in vitro uptake assays using recombinant HIF1A as a model substrate. We have previously demonstrated that HIF1A contains an active KFERQ motif (*15*, *18*). Incubation of ARPE-19 cells with the hypoxia mimetic compound CoCl_2_ showed that HIF1A is present in sEVs only when cells express LAMP2A (fig. S4A). Isolation of sEVs from 769-P cells, KO for HIF1A and the ubiquitin ligase Von Hippel–Lindau (*30*), and either rescuing HIF1A expression or expressing a KFERQ-mutant HIF1A (HIF1A^AA^) showed that only WT HIF1A is present in sEVs (fig. S4B). Moreover, subcellular fractioning using an OptiPrep linear gradient showed that endogenous HIF1A localizes to endosomal fractions and that LAMP2A KO decreases HIF1A presence in those fractions (fig. S4C). We next incubated endosomal fractions with glutathione *S*-transferase (GST) (no KFERQ motif) and HIF1A-GST, in the presence or absence of recombinant HSC70 and adenosine triphosphate (ATP), followed by trypsin treatment to degrade recombinant HIF1A protein unprotected by the endosomal membrane (Fig. 5A). Results show that only EE fractions incubated with HSC70 and ATP and containing LAMP2A protected HIF1A-GST from degradation by trypsin (Fig. 5A).

Confocal microscopy of cells fixed with methanol, to eliminate cytoplasmic mCherry and better resolve its vesicular localization (*31*), showed a high mCherry-ExoSignal colocalization coefficient with LAMP2A (76.67 ± 21.55%), as well as with early endosomal markers such as Rab5 (60.7 ± 29.58%) and EEA1 (84 ± 21.55%) (Fig. 5B and fig. S5A). However, we also observed low colocalization with the LE marker mannose 6-phosphate (m6p) receptor (12.8 ± 8.012%) (Fig. 5B and fig. S5A). In addition, LAMP2A showed low colocalization coefficient with m6p (22.44 ± 11.04%) and high colocalization with both CD63 (78.2 ± 9.52%) and EEA1 (89.33 ± 4.24%) (Fig. 5C). On the other hand, three-dimensional (3D) reconstruction of paraformaldehyde (PFA)–fixed ARPE-19 cells without permeabilization showed that LAMP2A was present at the plasma membrane, while LAMP2B was only residually present in the plasma membrane (fig. S5B). In accordance, functional enrichment analysis of the subcellular compartment localization of all proteins down-regulated by LAMP2A KO, with and without KFERQ motif, showed that LAMP2A KO leads to a significant down-regulation of proteins that are components of EEs (Fig. 5D). In addition, all down-regulated proteins associated with EEs contained the KFERQ motif (Fig. 5D).

Previous reports indicated that some proteins containing KFERQ-like motifs are degraded in endosomes by endosomal microautophagy (e-Mi) independently of LAMP2A (*32*). Incubation of two typical e-Mi substrates, namely, glyceraldehyde-3-phosphate dehydrogenase (GAPDH) and aldolase, with endosomes isolated from WT and LAMP2 KO cells showed that both recombinant proteins are resistant to trypsin digestion in EEs and LEs, independently of LAMP2A and HSC70 (fig. S5C).

Overall, these data indicate that LAMP2A, as well as HSC70, has a role in loading KFERQ-containing proteins into endosomal ILVs, suggesting that this mechanism is involved in the targeting of proteins into exosomes. Data also suggest that the targeting of KFERQ-containing proteins into endosomal ILVs begins early in the endocytic pathway.

### LAMP2A-mediated protein targeting to endosomes is independent of ESCRT machinery

**Fig. 6.**
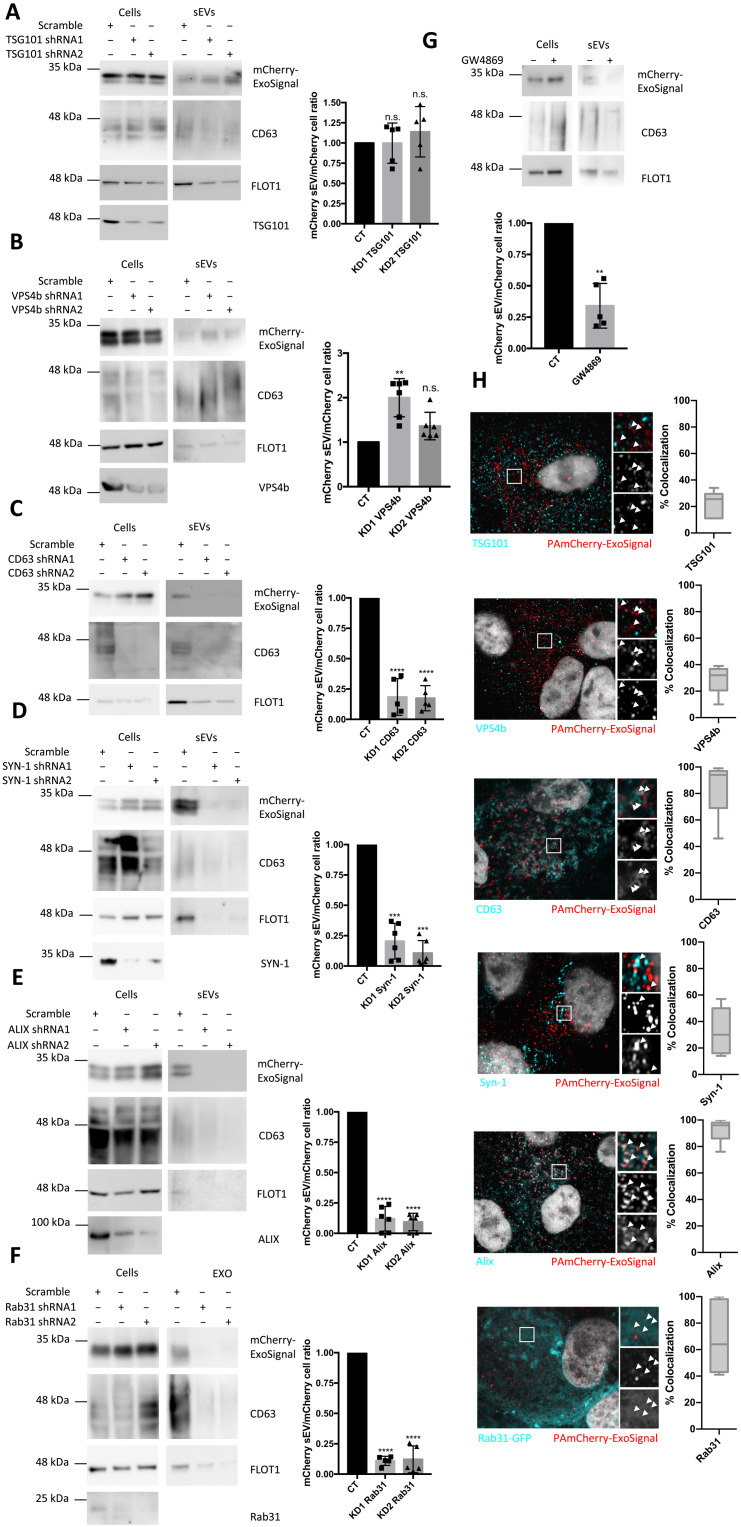
mCherry-ExoSignal is loaded into sEVs by ESCRT-independent mechanisms. WT ARPE-19 cells expressing mCherry-ExoSignal were transduced using lentiviral particles containing vectors for the expression of shRNAs. Cells were maintained in exosome-depleted medium for 48 hours. WB of exosomal fractions using antibodies against CD63, FLOT1, and mCherry. (**A**) TSG101 depletion decreases sEV secretion but has no impact on the levels of PAmCherry-ExoSignal present in isolated sEVs. (**B**) VPS4b depletion increases sEV secretion and the levels of PAmCherry-ExoSignal present in isolated sEVs. (**C** to **G**) CD63, Syntenin-1, Alix, and Rab31 depletion, as well as ceramide synthesis inhibition by GW4869, decreased sEV secretion and mCherry-ExoSignal levels in the vesicles. (**H**) Confocal images of methanol-fixated cells show high colocalization coefficient of mCherry-ExoSignal with CD63, Alix, and Rab31 and low colocalization coefficient for TSG101, VPS4b, and Syntenin-1. All samples were analyzed under the same experimental conditions. The results represent means ± SD of at least *n* = 3 independent experiments (n.s., nonsignificant; **P < 0.01, ***P < 0.001, and ****P < 0.0001).

TSG101 and VPS4b are components of the ESCRT-I and ESCRT-III machinery that are involved in ILV formation and exosome biogenesis (*33*). In addition, the mechanism of e-Mi is dependent on TSG101 and VPS4b (*32*). By depleting TSG101, we were able to observe a decrease in the levels of CD63 and Flotillin-1 in sEV fractions, indicating an inhibition in sEV release. However, the levels of mCherry-ExoSignal present in sEVs from control or TSG101-depleted cells were unaffected (Fig. 6A and fig. S6). On the contrary, VPS4b depletion increased the levels of CD63 and Flotillin-1 in sEVs (Fig. 6B and fig. S6). This is consistent with previous reports that VPS4b depletion led to an increase in exosome release (*33*). Accordingly, the levels of mCherry-ExoSignal present in sEVs after VPS4b depletion were also increased (Fig. 6B and fig. S6).

We next targeted ESCRT-independent mechanisms of exosome biogenesis (*33*), including the sphingolipid ceramide (*8*), the tetraspanin CD63 (*34*), the protein complex syndecan–Syntenin-1–Alix (*9*), and Rab31 (*35*). Depletion of CD63, Syntenin-1, Alix, and Rab31 using short hairpin RNA (shRNA) and depletion of ceramide levels using an inhibitor of ceramide synthesis (GW4869) inhibited sEV secretion and mCherry-ExoSignal presence in the sEV fraction (Fig. 6, C to G, and fig. S6). Confocal microscopy further showed low colocalization between mCherry-ExoSignal and either TSG101 (22.5 ± 9.439%) or VPS4b (28.83 ± 10.59%) (Fig. 6H and fig. S6). Conversely, images showed high colocalization with CD63 (83.36 ± 18.96%), Alix (92.33 ± 8.093%), and Rab31 (70.11 ± 26.49%) while indicating lower-than-expected colocalization with Syntenin-1 (31.45 ± 16.28%) (Fig. 6H and fig. S6).

**Fig. 7.**
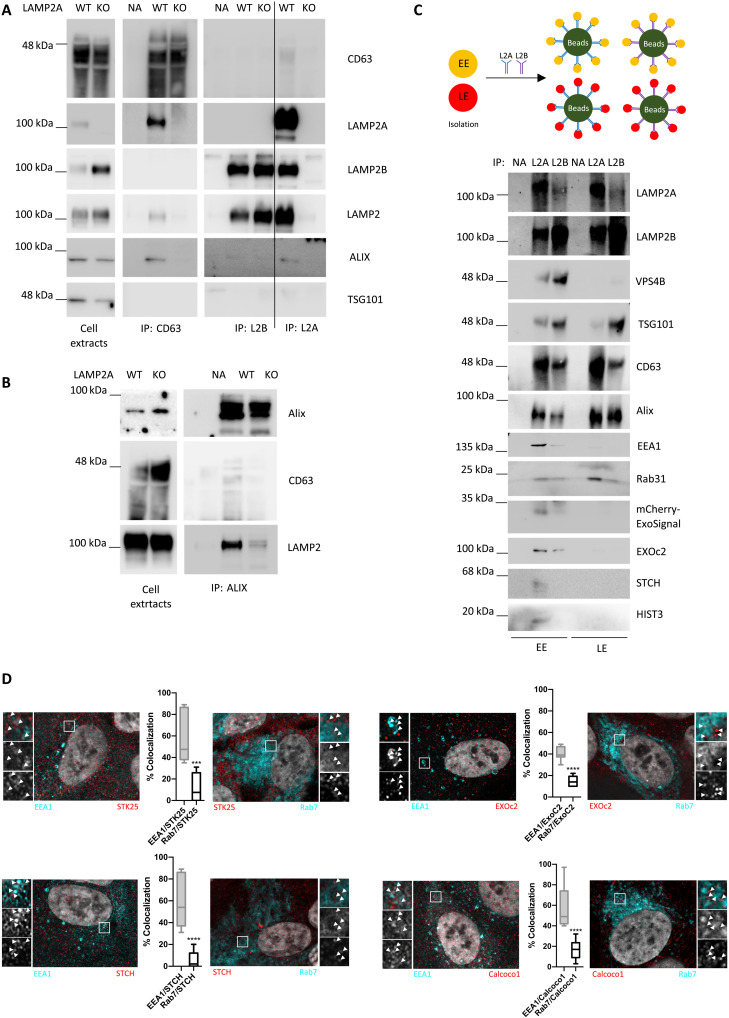
LAMP2A associates more closely to ESCRT-independent endosomal machinery. (**A** and **B**) Immunoprecipitation experiments using antibodies raised against LAMP2, LAMP2A, LAMP2B, CD63, Alix, and TSG101 show that LAMP2A, but not LAMP2B, coprecipitates with CD63 and Alix. TSG101 does not coprecipitate with either the LAMP2A or LAMP2B isoform. (**C**) Top: Schematic workflow of the experiment. EEs and LEs were further immunoprecipitated with antibodies raised against the LAMP2A or LAMP2B isoforms. WB shows (bottom) that VPS4B and TSG101 are enriched in LAMP2B pulled-down endosomes, while CD63; Alix; RAB31; mCherry-ExoSignal; and KFERQ motif–containing proteins EXOc2, STCH, and histone 3 are enriched in LAMP2A-enriched endosomes. (**D**) Confocal images show high colocalization coefficient of KFERQ motif–containing proteins EXOc2, STCH, STK25 (Serine/Threonine Kinase 25), and Calcoco1 with the EE marker EEA1 rather than with the LE marker Rab7. The results represent the mean ±SD of at least *n* = 3 independent experiments (n.s., nonsignificant; ***P < 0.001; ****P < 0.0001).

Next, we immunoprecipitated LAMP2A or LAMP2B using antibodies raised against the cytoplasmic tail of each specific isoform. Results show substantial cross-reactivity between the so-called LAMP2B-specific antibody and the LAMP2A protein isoform (Fig. 7A). No such cross-reaction is apparent with the LAMP2A-specific antibody (Fig. 7A). Nonetheless, we were able to coprecipitate CD63 and ALIX, but not TSG101, after LAMP2A immunoprecipitation and vice versa (Fig. 7, A and B). Conversely, in samples incubated with the less-specific anti-LAMP2B antibody, both CD63 and ALIX showed residual coprecipitation (Fig. 7A).

Subsequently, we isolated endosomes and immunoprecipitated the subcellular compartments with either anti-LAMP2A or anti-LAMP2B antibodies. When comparing LAMP2A with LAMP2B endosomes, the distribution of the different components was asymmetric. Higher levels of TSG101 and VPS4b were detected in endosome fractions enriched in LAMP2B, and higher levels of CD63 and Rab31 were found in endosome fractions enriched in LAMP2A (Fig. 7C). Alix was predominantly distributed toward LAMP2A endosomes only in EE-enriched fractions (Fig. 7C). Accordingly, mCherry-ExoSignal levels were higher in EEs enriched in LAMP2A (Fig. 7C), as were the levels of EXOc2 (Exocyst complex component 2), STCH (Stress-70 Protein Chaperone Microsome-Associated 60 KDa), and histone 3, proteins that contain KFERQ motifs and that were randomly selected from the down-regulated proteins in sEV MS data (Fig. 7C). Confocal microscopy further demonstrated that proteins selected from the same MS data preferentially colocalize with the EE marker EEA1 rather than with the LE marker Rab7 (Fig. 7D). In addition, images also showed that LAMP2B colocalization with LAMP2A is lower than expected (73.88 ± 13.09%), indicating that both LAMP2 isoforms are often segregated into different vesicles (fig. S6C). Overall, our evidence indicates that LAMP2A, but not LAMP2B, participates in the loading of proteins containing KFERQ-like motifs at the early endosomal membrane in association with CD63, Alix, Syntenin-1, Rab31, and ceramides.

### ExoSignal-tagged proteins are sorted into circulating exosomes in zebrafish larvae

**Fig. 8.**
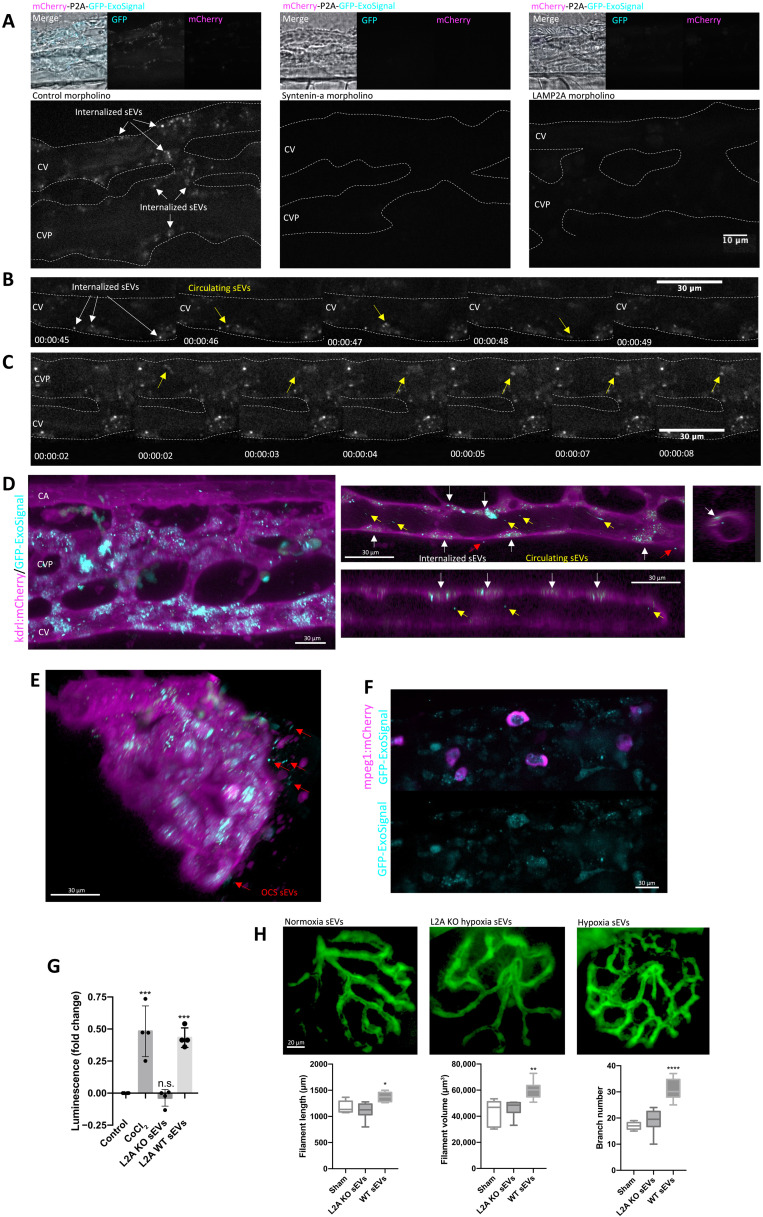
LAMP2A mediates loading of KFERQ-containing proteins into sEVs in the zebrafish larvae. Images and movies of the zebrafish show the caudal artery (CA), the caudal vein (CV), and a complex venous vascular network [caudal vascular plexus (CVP)] between CV and CA. (**A**) Casper zebrafish at 1000 cell stage were coinjected at the YSL with the GFP-ExoSignal-P2A-mCherry construct with either control, Syntenin-a, or LAMP2A MOs. Images show that GFP-ExoSignal is present in the CVP at 3 days postfertilization (dpf). Syntenin-a or LAMP2A MO inhibits GFP-ExoSignal circulation in the CVP. (**B** and **C**) Time-lapse of movies S6 and S7 showing GFP-ExoSignal sEVs in the CVP, with white arrows indicating incorporated sEVs and yellow arrows indicating circulating sEVs. (**D** and **E**) 3D reconstruction and orthogonal view of *Tg(kdrl:mCherry)* larvae, expressing mCherry in endothelial cells, injected with GFP-ExoSignal construct. Red arrows indicate outside circulatory network (OCS) sEVs. sEVs labeled with GFP-ExoSignal are present inside endothelial cells, in the vessel’s lumen and outside the OCS. (**F**) *Tg(mpeg1:mCherry)* larvae, expressing mCherry in macrophages. GFP-ExoSignal is present in macrophages located in the CVP. (**G**) HeLa cells expressing luciferase under the control of a hypoxia response element (HRE) were incubated with WT (loaded with HIF1A) and LAMP2A KO (missing HIF1A) sEVs. HIF1A from sEVs activates luciferase expression. (**H**) A total of 0.25 μg of isolated WT (loaded with HIF1A) and LAMP2A KO (missing HIF1A) sEVs was injected in 3 dpf zebrafish embryos expressing GFP in endothelial cells *Tg(fli1a:EGFP)*. At 5 dpf, whole-mounted embryos were imaged. sEVs loaded with HIF1A are able to induce neovascularization in the zebrafish embryos. All samples were analyzed under the same experimental conditions. 3D reconstruction of the images and quantifications were performed using Imaris software. The results represent means ± SD of at least *n* = 3 independent experiments (*P < 0.05, **P < 0.01, ***P < 0.001, and ****P < 0.0001).

In a recent report, we followed circulating exosomes in zebrafish larvae as they traveled from one organ to another using CD63 tagged with a fluorescent protein (*36*). Building from that conceptual work, we created a new construct composed of untagged mCherry, which also contains the self-cleaving porcine teschovirus-1–derived peptide (P2A) (*37*) and GFP tagged with the ExoSignal (fig. S7A). We injected the yolk syncytial layer (YSL) of zebrafish embryos, a highly secretory and irrigated organ of the zebrafish larvae (*36*), and imaged the zebrafish caudal plexus, located posteriorly to the yolk sack (fig. S7A). When compared with sham-injected larvae or untagged GFP, mCherry-P2A-GFP-ExoSignal–injected larvae were positive for GFP in the caudal plexus, while mCherry signal was residual (Fig. 8A, fig. S7B, and movies S1 to S5). GFP-ExoSignal–positive puncta, likely representing exosomes, circulated in the bloodstream (Fig. 8B and movies S1 to S3 and S6 in higher magnification) and often stopped at the vessel wall (Fig. 8C and movie S7). Moreover, sEVs isolated from zebrafish larvae (*36*) contained GFP-ExoSignal but not mCherry (fig. S7C). To inhibit exosome secretion, zebrafish were coinjected with a morpholino (MO) raised against zebrafish syntenin-a (fig. S7D) (*36*, *38*), the zebrafish homolog for human syntenin-1, as well as an MO against zebrafish LAMP2A to inhibit the loading of proteins into exosomes. Coinjection of both MOs did not change the overall expression of GFP-ExoSignal or CD63 in zebrafish larvae (fig. S7E) but led to a decrease in GFP-ExoSignal in the caudal plexus (Fig. 8A and movies S8 and S9).

3D reconstruction of the caudal plexus of *Tg(kdrl:mCherry)*, which expresses mCherry in endothelial cells, showed that GFP-ExoSignal was present in the lumen of the blood vessels and inside endothelial cells (Fig. 8D), while no GFP signal was present in endothelial cells under the sham-injected or syntenin-a MO conditions (fig. S7F). In some instances, GFP also localized outside the vascular network (Fig. 8E). By live imaging of *Tg(mpeg1:mCherry)*, which expresses mCherry in macrophages, we observed that macrophages moving within the caudal plexus incorporated GFP-ExoSignal (Fig. 8F, fig. S7G, and movies S10 and S11). Overall, our data show that EVs, likely exosomes, loaded with GFP-ExoSignal are released from the YSL into the circulation, traveling through the bloodstream to reach the caudal plexus, where they are incorporated by endothelial cells and infiltrating macrophages.

### LAMP2A regulates intercellular transfer of hypoxia signaling through the HIF1A transcription factor

We next incubated 769-P cells, which are KO for HIF1A (*30*), with sEVs loaded with HIF1A. Subcellular fractioning of 769-P cells showed that HIF1A transported by sEVs reached the cytosol and the nucleus (fig. S7H). HeLa cells expressing an HIF1A reporter gene with luciferase under the control of a hypoxia response element (HRE) consensus sequence and incubated with WT (loaded with HIF1A) and LAMP2A KO (lacking HIF1A) sEVs showed that only sEVs secreted by LAMP2A WT cells induced luciferase expression (Fig. 8G).

HIF1A adaptive response to hypoxia involves the formation of new blood vessels or neovascularization (*39*). By injecting a zebrafish transgenic line *Tg(fli1a:EGFP)*, expressing GFP in endothelial cells, with sEVs from WT cells, we were able to increase the number of vascular branches, including longer blood vessels with greater volume, in the retinal vascular network (Fig. 8H), while sEVs isolated from LAMP2A KO cells failed to do so. Overall, these data indicate that cells expressing LAMP2A release exosomes carrying HIF1A that are capable of reaching receiving cells long distances away from the originating cells. These exosomes secreted by hypoxic cells can activate a hypoxia response, likely mediated by HIF1A.

## DISCUSSION

Here, we propose a mechanism whereby LAMP2A, in coordination with HSC70 and additional molecular players, participates in the loading of cytosolic proteins into a subpopulation of exosomes (fig. S8). The targeted proteins contain amino acid sequences, biochemically related to the KFERQ motif, a pentapeptide sequence previously involved in two selective forms of autophagy: chaperone-mediated autophagy (CMA) (*15*, *40*) and e-Mi (*32*). LAMP2A was initially thought to be involved exclusively in CMA by mediating substrate translocation across the lysosomal membrane in a process that was dependent on the chaperone HSC70 (*15*, *40*). We now show that LAMP2A KO significantly down-regulated 303 proteins from secreted sEVs, of which ~^2^/_3_ contain at least one KFERQ motif. While we do not know the specific mechanisms that regulate the remaining ~^1^/_3_ of down-regulated proteins that do not contain KFERQ motifs, it is conceivable that they represent membrane proteins that interact with LAMP2A at the endosomal membrane and/or proteins that are in a complex with KFERQ-containing proteins while these are being loaded into nascent ILVs. In this context, we suggest that LAMP2A participates primarily in the loading of proteins into ILVs at the endosomal membrane. While sEV fractions isolated by ultracentrifugation at 120,000*g* contain both small microvesicles, originating from the shedding of the plasma membrane, and vesicles of endosomal origin, known as exosomes, our data show that proteins containing KFERQ motifs, in the presence of LAMP2A, are loaded into nascent ILVs. The endosomal localization of such proteins is a compelling piece of evidence indicating that LAMP2A participates in the loading of proteins into exosomes. Our data also suggest that endosomal LAMP2A does not participate in the events leading to ILV formation by the inward budding of the endosomal limiting membrane. Instead, it is likely that by binding to HSC70 and associated KFERQ-containing proteins, LAMP2A captures its cargo during ILV biogenesis.

In addition, our results show that the loading of proteins containing KFERQ motifs into endosomes is likely restricted to EEs. This is expected since it is possible that the ability to generate ILVs is greater in EEs than in LEs. For example, it was shown that CD63 is ~7× enriched in the ILVs of LEs as compared to the endosomal limiting membrane (*41*). Nonetheless, it is intriguing that the loading of the two e-Mi substrates GAPDH and aldolase, which were described to be independent of LAMP2A (*32*), occurs both in EE- and LE-enriched fractions. In this case, it is likely that the machinery associated to e-Mi, including the ESCRT machinery (*32*), is active in later endosomal compartments, while LAMP2A and other ESCRT-independent machinery are not. Further research is needed to clarify these discrepancies.

Our data indicate that LAMP2A-mediated loading of mCherry-ExoSignal into exosomes is ESCRT independent and also involves CD63, ALIX, Syntenin-1, Rab31, and ceramides. After the pulldown of endosomes enriched in either the LAMP2A or LAMP2B isoforms, the different endosomal components were asymmetrically segregated, with the ESCRT machinery preferentially localizing to LAMP2B-enriched endosomes, while the other components segregated preferentially to LAMP2A-enriched endosomes. This supports a model in which endosomal compartments within a cell may contain distinct molecular machineries, which will affect the final contents of ILVs and, as a consequence, of exosomes.

To assess the biological impact of LAMP2A-mediated exosomal loading, we used the zebrafish model. Only when GFP was tagged with the ExoSignal were we able to observe exosomes being transferred from the YSL to endothelial cells and macrophages within the caudal plexus via circulation. Both depletion of exosome secretion by using the syntenin-a MO (*36*) and using an MO for LAMP2A were able to inhibit the presence of circulating GFP-ExoSignal, further corroborating our in vitro observations.

Our laboratory had previously reported that the transcription factor HIF1A is a substrate for CMA (*15*, *42*). Data presented here show that HIF1A is selectively targeted to exosomes by the virtue of its KFERQ motif and LAMP2A. Exosomes loaded with HIF1A can activate HIF1A signaling in normoxic cells in vitro, while, in the zebrafish animal model, exosomes isolated from hypoxic cells can activate neovascularization, likely by the action of exosomal HIF1A. However, we cannot exclude that other proteins that were up- or down-regulated following LAMP2A KO may somewhat account for the effects observed in angiogenesis.

To summarize, in this study, we propose that LAMP2A mediates the loading of proteins into exosomes in a process that is ESCRT independent and involves HSC70 recognition of active KFERQ motifs. Endosomal proteins such as CD63, Syntenin-1, Alix, Rab31, and ceramides appear to be required for the selective loading, although their roles are not yet clear. Evidence also suggests that this mechanism may affect intercellular and interorgan communication by promoting the loading of the hypoxia transcription factor HIF1A into exosomes with the potential to activate hypoxia response in distant normoxic cells and tissues.

## METHODS

### Cell culture and treatments

The human cell lines ARPE-19 (originally derived from retinal pigment epithelium) and 769-P (renal cell adenocarcinoma) were cultured in Dulbecco’s modified Eagle’s medium (DMEM) with glutamine (Biowest) supplemented with 10% fetal bovine serum (FBS; Biowest) and penicillin-streptomycin (100 U/ml:100 μg/ml; Gibco). Cells were cultured at 37°C under 5% CO_2_. When appropriate, cells were treated with the following agents: 300 μM cobalt chloride (CoCl_2_; Sigma-Aldrich), 7.5 μM Pifi (Merck), and 50 nM bafilomycin A1 (Apollo Scientific).

### Antibodies and reagents

The following antibodies were used: mouse anti-LAMP2 clone H4B4, dilution of 1:1000 (WB) and 1:100 (immunofluorescence; Santa Cruz Biotechnology, SC756); rabbit anti-LAMP2A, dilution of 1:500 (WB) and 1:100 (immunofluorescence; Abcam, ab18528); rabbit anti-LAMP2B, dilution of 1:500 (Abcam, ab18529); mouse anti-actin, dilution of 1:2000 (Sigma-Aldrich, AS441); goat anti-HIF1A, dilution of 1:1000 (SICGEN, AB0112-200); goat anti-CD63, dilution of 1:1000 (SICGEN, AB0047-200); mouse anti-CD63, dilution of 1:100 (immunofluorescence; Santa Cruz Biotechnology, MX-49.129.5); rabbit anti–Flotillin-1, dilution of 1:500 (Santa Cruz Biotechnology, H-104); goat anti-CTSB/cathepsin B clone S-12, dilution of 1:500 (Santa Cruz Biotechnology, SC-6493); mouse anti-Alix, dilution of 1:500 (Santa Cruz Biotechnology, sc-53540); mouse anti-TSG101, dilution of 1:500 (GeneTex, GTX70255); goat anti-GST, dilution of 1:500 (SICGEN, AB9919-500); goat anti-EEA1, dilution of 1:500 (SICGEN, AB0006-200); goat anti-mCherry, dilution of 1:500 (WB) and 1:100 (immunofluorescence; SICGEN, AB0040-200); rabbit anti-DsRed, dilution of 1:100 (immunofluorescence; Clontech, 632496); mouse anti-m6pR, dilution of 1:100 (immunofluorescence; Abcam, ab2733); rat anti-HSC70 clone 1B5, dilution of 1:1000 (Stressgen, ADI-SPA-815); goat anti-Rab27, dilution of 1:500 (SICGEN, AB7223-200); goat anti-tubulin, dilution of 1:2000 (SICGEN, AB0046-200); mouse lamin B1, dilution of 1:500 (Santa Cruz Biotechnology, sc-374015); goat anti-calnexin, dilution of 1:1000 (SICGEN, AB0041-200); goat anti–Na^+^,K^+^-ATPase, dilution of 1:1000 (SICGEN, AB0306-200); goat anti-GAPDH, dilution of 1:2000 (SICGEN, AB0049-200); mouse anti-ExoC2, dilution of 1:500 (Novus Biological, NBP1-83786); rabbit anti–histone 3, dilution of 1:1000 (Sigma-Aldrich, H0164); anti-Vps4b, dilution of 1:500 (Santa Cruz Biotechnology, sc-133122), goat anti-GFP, dilution of 1:1000 (SICGEN, AB0020-200), goat anti-Rab31, dilution of 1:500 (SICGEN, AB0068-200); mouse anti–syntenin-1 (Santa Cruz Biotechnology, sc-515538); and horseradish peroxidase (HRP)–conjugated secondary goat anti-mouse (Bio-Rad, 170-6516), goat anti-rabbit (Bio-Rad, 170-6515), rabbit anti-goat (Bio-Rad, 172-1034), and goat anti-rat (Invitrogen, A10549), dilution of 1:5000. The following were also used: Alexa Fluor 568–conjugated donkey anti-goat (Invitrogen, A11057), Alexa Fluor 488–conjugated donkey anti-goat (Invitrogen, A32814), Alexa Fluor 488–conjugated donkey anti-rabbit (Invitrogen, A32790), Alexa Fluor 546–conjugated donkey anti-mouse (Invitrogen, A10036), donkey anti-rabbit Cy3 (Abcam, ab6566), and donkey anti-mouse Cy5, dilution of 1:250. The following were also used: dextran, Alexa Fluor 488, 10,000 molecular weight (MW), anionic, fixable (Thermo Fisher Scientific, D22910); dextran, Alexa Fluor 647, 10,000 MW, anionic, fixable (Thermo Fisher Scientific, D22914); wheat germ agglutinin, Alexa Fluor 488 conjugate (Thermo Fisher Scientific, W11261); cathepsin B assay kit (Magic Red; Abcam, ab270772); DAPI (4′,6-diamidino-2-phenylindole, dihydrochloride; Invitrogen, D1306); Protein G–Sepharose (GE Healthcare, 17-0618-01); Dynabeads Protein G (Invitrogen, 10004B); nitrocellulose membranes (GE Healthcare, 88018); ECL (GE Healthcare, RPN1235); protease inhibitor cocktail (Sigma-Aldrich, P8340); OptiPrep iodixanol density media (Sigma-Aldrich, D1556); ONE-Glo Luciferase Assay System (Promega, E6110); and Pierce BCA Protein Assay Kit (Thermo Fisher Scientific, 23225).

### Peptide sample preparation for MS

The sEV protein solution containing SDS and dithiothreitol (DTT) was loaded onto filtering columns and washed exhaustively with 8 M urea in Hepes buffer (*43*). Proteins were reduced with DTT and alkylated with indole-3-acetic acid. Protein digestion was performed by overnight digestion with trypsin sequencing grade (Promega).

### Nano–LC-MS/MS analysis

Peptide samples were analyzed by nano–LC-MS/MS (Dionex RSLCnano 3000) coupled to a Q-Exactive Orbitrap mass spectrometer (Thermo Scientific). Briefly, the samples (5 μl) were loaded onto a custom-made fused capillary precolumn (length, 2 cm; outer diameter, 360 μm; and inner diameter, 75 μm) with a flow of 5μl/min for 7 min. Trapped peptides were separated on a custom-made fused capillary column (length, 20 cm; outer diameter, 360 μm, and inner diameter, 75 μm) packed with ReproSil-PurC18 3-μm resin (Dr. Maisch, Ammerbuch-Entringen, Germany) with a flow of 300 nl/min using a linear gradient from 92% A (0.1% formic acid) to 28% B (0.1% formic acid in 100% acetonitrile) over 93 min followed by a linear gradient from 28 to 35% B over 20 min at a flow rate of 300 nl/min. Mass spectra were acquired in positive ion mode, applying automatic data-dependent switch between one Orbitrap survey. MS scan in the mass range of 400 to 1200 mass/charge ratio (*m*/*z*) was followed by higher-energy collisional dissociation (HCD) fragmentation and Orbitrap detection of the 15 most intense ions observed in the MS scan. Target value in the Orbitrap for MS scan was 1,000,000 ions at a resolution of 70,000 at *m*/*z* 200. Fragmentation in the HCD cell was performed at normalized collision energy of 31 eV. Ion selection threshold was set to 25,000 counts, and maximum injection time was 100 ms for MS scans and 300 and 500 ms for MS/MS scans. Selected sequenced ions were dynamically excluded for 45 s.

### MS database search

The obtained data from the 36 LC-MS runs were searched using VEMS (*44*, *45*) and MaxQuant (*46*). A standard proteome database from UniProt (3AUP000005640), in which common contaminants were included, was also searched. Trypsin cleavage allowing a maximum of four missed cleavages was used. Carbamidomethyl cysteine was included as fixed modification. Methionine oxidation; N-terminal protein acetylation; lysine acetylation; lysine diglycine; and S, T, and Y phosphorylation were included as variable modifications; mass accuracy of 10 parts per million was specified for precursor ions and 0.01 *m*/*z* for fragment ions. The false discovery rate for protein identification was set to 1% for peptide and protein identifications. No restriction was applied for minimal peptide length for VEMS search. Identified proteins were divided into evidence groups as defined (*44*).

### MS data analysis

Statistical and bioinformatics analyses of MS data were performed in the statistical programming language R. Quantitative data from MaxQuant and VEMS were analyzed in R statistical programming language. IBAQ and protein spectral counts from the two programs were preprocessed by three approaches: (i) removing common MS contaminants followed by log_2_ (*x* + 1) transformation; (ii) removing common MS contaminants followed by log_2_ (*x* + 1) transformation and quantile normalization; and (iii) removing common MS contaminants followed by log_2_ (*x* + 1) transformation, quantile normalization, and abundance filtering to optimize overall Gaussian distribution of the quantitative values. For simplicity, only the quantile-normalized quantitative data are presented here. Statistical differences were calculated by using the R package limma (*47*). Correction for multiple testing was applied using the Benjamini-Hochberg method (*48*). Comparison of EV markers and potential contaminants in the current study with those in a previous study was performed as previously described (*19*). Functional enrichment of Kyoto Encyclopedia of Genes and Genomes, cellular component, molecular function, and biological process were calculated by using the hypergeometric probability test as previously described (*49*, *50*). For the search of KFERQ-like motifs, we used the online tool KFERQ finder (https://rshine.einsteinmed.org/).

### Plasmids for transfection

For this work, we used the plasmids pT81 HRE (x3)-Luciferase (*51*) and pEGFP-Rab5QL.

### Clustered regularly interspaced short palindromic repeats–Cas9

ARPE-19 cells were transduced with the lentiviral vector pCW-Cas9 (a gift from E. Lander and D. Sabatini; Addgene plasmid #50661; http://n2t.net/addgene:50661; RRID: Addgene_50661) (*52*) for doxycycline-induced expression of SpCas9. Infected cells were selected with puromycin. Cells were allowed to grow in very low confluency, and a one-cell colony was selected and allowed to reach confluency. The selected isotype was used from this point on. The guide RNA (gRNA) targeting LAMP2A (5′-AGTACTTATTCTAGTGTTGC-3′) was expressed under U6 promoter. The expression cassette contained the U6 promoter, target sequence, PAM sequence, gRNA, and the termination signal for polymerase III (Pol III). This sequence was flanked by attB1/attB2, synthesized by GeneCust, and subcloned into pLenti6, containing blasticidin resistance (Thermo Scientific), with a Gateway adapted vector using BP/LR Clonase II (Thermo Scientific), according to the manufacturer’s instructions. The guide was designed using the online Guide Design Resources tool from Zhang Lab (https://zlab.bio/guide-design-resources).

For the KO of LAMP2A isoform, cells were incubated with doxycycline for 48 hours and were subsequently allowed to grow in very low confluency. Individual colonies were allowed to reach confluency, and the KO of LAMP2A was confirmed by DNA sequencing and WB.

### Lentiviral plasmids for protein expression

All lentiviral particles were produced by cotransfection of pLenti6 and pMD 2.G [vesicular stomatitis virus glycoprotein (VSV-G) protein] and psPAX2 (Rev and Pol proteins) into a producer cell line, 293STAR RDPro [American Type Culture Collection (ATCC)]. Recombinant viral particles were harvested 48 days later, cleared for cell debris by centrifugation at 3200*g* for 10 min, and used. pLenti6-wtHIF1A and KFERQ-mutated pLenti6-AAHIF1A (*15*) were obtained from the Molecular Biology Platform (CEDOC, Lisbon). PA-mCherry and PA-mCherry ExoSignal were flanked by attB1/attB2 Gateway (Thermo Scientific) with the sequence 5′-ATGGTGAGCAAGGGCGAGGAGGATAACATGGCCATCATTAAGGAGTTCATGCGCTTCAAGGTGCACATGGAGGGGTCCGTGAACGGCCACGTGTTCGAGATCGAGGGCGAGGGCGAGGGCCGCCCCTACGAGGGCACCCAGACCGCCAAGCTGAAGGTGACCAAGGGCGGCCCCCTGCCCTTCACCTGGGACATCCTGAGCCCTCAGTTCATGTACGGCTCCAATGCCTACGTGAAGCACCCCGCCGACATCCCCGACTACTTTAAGCTGTCCTTCCCCGAGGGCTTCAAGTGGGAGCGCGTGATGAAATTCGAGGACGGCGGCGTGGTGACCGTGACCCAGGACTCCTCCCTGCAGGACGGCGAGTTCATCTACAAGGTGAAGCTGCGCGGCACCAACTTCCCCTCCGACGGCCCCGTGATGCAGAAGAAGACCATGGGCTGGGAGGCCCTCTCCGAGCGGATGTACCCCGAGGACGGCGCCCTGAAGGGCGAGGTCAAGCCCAGAGTGAAGCTGAAGGACGGCGGCCACTACGACGCTGAGGTCAAGACCACCTACAAGGCCAAGAAGCCCGTGCAGCTGCCCGGCGCCTACAACGTCAACCGCAAGCTGGACATCACCAGCCACAACGAGGACTACACCATCGTGGAGCAGTACGAGAGAGCCGAGGGCCGCCACTCCACCGGCGGCATGGACGAGCTGTACAAG-3′.

The sequences were synthesized and cloned into pUC57 (GeneCust). PA-mCherry and PA-mCherry ExoSignal were subcloned into pLenti6 (Thermo Scientific) using a Gateway adapted vector using BP/LR Clonase II (Thermo Scientific) according to the manufacturer’s instructions.

Human LAMP2A was synthesized into pUC57 plasmid with the sequence 5′-CAAGTTTGTACAAAAAAGCAGGCTCTCGAGCAatgGTGTGCTTCCGCCTCTTCCCGGTTCCGGGCTCAGGGCTCGTTCTGGTCTGCCTAGTCCTGGGAGCTGTGCGGTCTTATGCATTGGAACTTAATTTGACAGATTCAGAAAATGCCACTTGCCTTTATGCAAAATGGCAGATGAATTTCACAGTACGCTATGAAACTACAAATAAAACTTATAAAACTGTAACCATTTCAGACCATGGCACTGTGACATATAATGGAAGCATTTGTGGGGATGATCAGAATGGTCCCAAAATAGCAGTGCAGTTCGGACCTGGCTTTTCCTGGATTGCGAATTTTACCAAGGCAGCATCTACTTATTCAATTGACAGCGTCTCATTTTCCTACAACACTGGTGATAACACAACATTTCCTGATGCTGAAGATAAAGGAATTCTTACTGTTGATGAACTTTTGGCCATCAGAATTCCATTGAATGACCTTTTTAGATGCAATAGTTTATCAACTTTGGAAAAGAATGATGTTGTCCAACACTACTGGGATGTTCTTGTACAAGCTTTTGTCCAAAATGGCACAGTGAGCACAAATGAGTTCCTGTGTGATAAAGACAAAACTTCAACAGTGGCACCCACCATACACACCACTGTGCCATCTCCTACTACAACACCTACTCCAAAGGAAAAACCAGAAGCTGGAACCTATTCAGTTAATAATGGCAATGATACTTGTCTGCTGGCTACCATGGGGCTGCAGCTGAACATCACTCAGGATAAGGTTGCTTCAGTTATTAACATCAACCCCAATACAACTCACTCCACAGGCAGCTGCCGTTCTCACACTGCTCTACTTAGACTCAATAGCAGCACCATTAAGTATCTAGACTTTGTCTTTGCTGTGAAAAATGAAAACCGATTTTATCTGAAGGAAGTGAACATCAGCATGTATTTGGTTAATGGCTCCGTTTTCAGCATTGCAAATAACAATCTCAGCTACTGGGATGCCCCCCTGGGAAGTTCTTATATGTGCAACAAAGAGCAGACTGTTTCAGTGTCTGGAGCATTTCAGATAAATACCTTTGATCTAAGGGTTCAGCCTTTCAATGTGACACAAGGAAAGTATTCTACAGCTCAAGACTGCAGTGCAGATGACGACAACTTCCTTGTGCCCATAGCGGTGGGAGCTGCCTTGGCAGGAGTACTTATTCTAGTGTTGCTGGCTTATTTTATTGGTCTCAAGCACCATCATGCTGGATATGAGCAATTTTAGGGTACCACCCAGCTTTCTTGTACAAAGTGGACGCGT-3′. Human LAMP2A was subcloned into pLenti6 (Thermo Scientific), a Gateway adapted vector using BP/LR Clonase II (Thermo Scientific), according to the manufacturer’s instructions.

P2A-GFP-ExoSignal was synthesized and subcloned into the p3E plasmid with the sequence 5′-TACAGGTCACTAATACCATCTAAGTAGTTGATTCATAGTGACTGCATATGTTGTGTTTTACAGTATTATGTAGTCTGTTTTTTATGCAAAATCTAATTTAATATATTGATATTTATATCATTTTACGTTTCTCGTTCAACTTTCTTGTACAAAGTGGAGATCTatgGGAAGCGGACGTACTAACTTCAGCCTGCTGAAGCAGGCTGGAGACGTGGAGGAGAACCCTGGACCTGCTCGAGCAGTGAGCAAGGGCGAGGAGCTGTTCACCGGGGTGGTGCCCATCCTGGTCGAGCTGGACGGCGACGTAAACGGCCACAAGTTCAGCGTGTCCGGCGAGGGCGAGGGCGATGCCACCTACGGCAAGCTGACCCTGAAGTTCATCTGCACCACCGGCAAGCTGCCCGTGCCCTGGCCCACCCTCGTGACCACCCTGACCTACGGCGTGCAGTGCTTCAGCCGCTACCCCGACCACATGAAGCAGCACGACTTCTTCAAGTCCGCCATGCCCGAAGGCTACGTCCAGGAGCGCACCATCTTCTTCAAGGACGACGGCAACTACAAGACCCGCGCCGAGGTGAAGTTCGAGGGCGACACCCTGGTGAACCGCATCGAGCTGAAGGGCATCGACTTCAAGGAGGACGGCAACATCCTGGGGCACAAGCTGGAGTACAACTACAACAGCCACAACGTCTATATCATGGCCGACAAGCAGAAGAACGGCATCAAGGTGAACTTCAAGATCCGCCACAACATCGAGGACGGCAGCGTGCAGCTCGCCGACCACTACCAGCAGAACACCCCCATCGGCGACGGCCCCGTGCTGCTGCCCGACAACCACTACCTGAGCACCCAGTCCGCCCTGAGCAAAGACCCCAACGAGAAGCGCGATCACATGGTCCTGCTGGAGTTCGTGACCGCCGCCGGGATCACTCTCGGCATGGACGAGCTGTACAAGGAAAGCTTTGTTAAAAAAGACCAAGCAGAACCACTACACCGAAAATTCGAACGACAATGAGGTACCGTCGACCAACTTTATTATACAAAGTTGGCATTATAAAAAAGCATTGCTTATCAATTTGTTGCAACGAACAGGTCACTATCAGTCAAAATAAAATCATTA-3′. Subsequently, the sequence was subcloned into a pDestTol2pA5 plasmid (gift from C.-H. Yuhm’s laboratory) according to the manufacturer’s instructions (Thermo Scientific), along with p5E-Ubi (zebrafish ubiquitin promoter; gift from A. Jacinto’s laboratory) and pME-mCherry (gift from C.-H. Yuhm’s laboratory).

### Lentiviral plasmids for protein knockdown

All lentiviral particles were produced by cotransfection of pLenti6 and pMD 2.G (VSV-G protein) and psPAX2 (Rev and Pol proteins) into a producer cell line, 293STAR RDPro (ATCC). Recombinant viral particles were harvested 48 days later, cleared for cell debris by centrifugation at 3200*g* for 10 min, and used. For TSG101, VPS4b, CD63, and Alix, pLKO.1 plasmids with shRNA sequences were obtained from the RNAi Consortium (Broad Institute, Boston). Control used was a nontargeting sequence (Mission; 5′-CAACAAGATGAAGAGCACCAA-3′; Sigma-Aldrich). The target sequences used were as follows: TSG101 (KD1, 5′-GCCTTATAGAGGTAATACATA-3′; KD2, 5′-CGTCCTATTTCGGCATCCTAT-3′), VPS4b (KD1, 5′-GCTGATCCTAACCATCTTGTA-3′; KD2, 5′-CCATTGTTATAGAACGACCAA-3′), CD63 (KD1, 5′-TGGGATTAATTTCAACGAGAA-3′; KD2, 5′-GCTGGCTATGTGTTTAGAGAT-3′), and Alix (KD1, 5′-GCAGAACAGAACCTGGATAAT-3′; KD2, 5′-GCATCTCGCTATGATGAATAT-3′). For Syntenin-1 target, pLKO.1 plasmids with shRNA sequences were obtained from Sigma Mission library (KD1, 5′-CCTATCCCTCACGATGGAAAT-3′; KD2, 5′-GAGAAGATTACCATGACCATT-3′).

### Adenoviral Rab27 and RAB31 miRNA

For the knockdowns, Rab27a and Rab27b, as well as Rab31 microRNA (miRNA)–expressing vectors, were constructed by inserting specific nucleotide sequences into pcDNA6.2-GW/EmGFP-miR plasmid harboring a Pol II promoter obtained from Thermo Scientific. These sequences are fused with GFP coding sequence. The synthesized oligonucleotides were annealed and ligated into pcDNA6.2-GW/EmGFP-miR according to the manufacturers’ instructions. For Rab27a and Rab27b, tandem miRNA sequences were transferred into pAd adenoviral vector from Thermo Scientific using Gateway technology. Sequences used were as follows: Rab27a miRNA1, 5′-AAACTTTGCTCATTTGTCAGG-3′; Rab27a miRNA2, 5′-TTAACTGATCCGTAGAGGCAT-3′; Rab27b miRNA1, 5′-ATTGACTTCCCTCTGATCTGG-3′; and Rab27b miRNA2, 5′-TTTCCCTGAAGATCCATTCGG-3′. For Rab31, miRNA sequences were transferred into pAd adenoviral vector from Thermo Scientific using Gateway technology. Sequences used were as follows: miRNA1, 5′-TTTCTTTGCAGGAAACGTCCC-3′ and miRNA2, 5′-TAAACTGAAGGCCATGTTGCG-3′. Viral particles were produced, and cells were infected as described before (*51*).

### Transmission electron microscopy

For ultrathin cryosectioning, cells were fixed in 2% PFA and 0.2% glutaraldehyde (GA) in 0.1 M phosphate buffer (pH 7.4). Cells were processed for ultracryomicrotomy and contrasted as described (*53*). All samples were examined with a FEI Tecnai Spirit electron microscope (Thermo Fisher Scientific), and digital acquisitions were made with a numeric camera (Quemesa, EMSIS).

For exosomal samples, exosomes were fixed with 2% PFA and deposited on Formvar-carbon–coated grids (TAAB Laboratories). Samples were washed with phosphate-buffered saline (PBS) and fixed with 1% GA for 5 min. After washing with distilled water, grids were contrasted with uranyl oxalate (pH 7) for 5 min and transferred to methylcellulose-uranyl acetate for 10 min on ice. Observations were carried out using a Tecnai G2 Spirit BioTwin electron microscope (FEI) at 80 kV.

### Exosome isolation

Exosomes derived from 40 million cultured cells were isolated from 40 ml of conditioned medium. Cells were cultured in exosome-depleted medium for 48 hours, prepared in accordance with the work of Lasser and colleagues (*54*). After incubation, the medium was collected, and exosomes were isolated by ultracentrifugation, as described before (*54*). Briefly, the harvested supernatant was subjected to differential centrifugation at 4°C, starting with centrifugation at 300*g* for 10 min, followed by centrifugation at 16,500*g* for 20 min. From this pellet, the microvesicle fraction was collected. To remove larger particles, the supernatant was filtered with a 0.22-μm filter unit, after which it was ultracentrifuged at 120,000*g* for 70 min, using an SW 32 Ti rotor. The resulting pellet was washed with PBS, and after ultracentrifugation, exosomes were resuspendedin PBS.

For MS, an extra step of purification was introduced using a 30% sucrose cushion [adapted from the work of Thery *et al.* (*55*)] to eliminate sample contaminants. Exosomal pellet was resuspended in 30 ml of cold PBS, loaded on top of 4 ml of tris/sucrose/D2O solution, and ultracentrifuged for 75 min at 100,000*g* and 4°C. Tris/sucrose/D2O cushion (∼3.5 ml) containing the exosomes was collected using a syringe with a 18-gauge needle. Exosomes were then transferred to a new ultracentrifuge tube, diluted in 35 ml of PBS, and centrifuged overnight at 100,000*g* and 4°C. The resulting pellet was resuspended in 100 μl of PBS, and protein quantification was performed.

### Western blot

Cell extracts and EV samples were denaturated in Laemmli buffer and heated at 95°C for 5 min. For cell extracts, we used ~2.5 μg of protein, and for sEV samples, we used the total amount of isolated vesicles secreted from 40 million cells. Total protein was resolved on a 10% SDS gel and transferred to a nitrocellulose membrane for 75 min at 100 V. The membranes were blocked with tris-buffered saline with 0.1% Tween 20 (TBS-T) containing 5% skim milk, followed by incubation with primary antibodies, overnight at 4°C. Membranes were incubated with HRP-conjugated secondary antibodies for 1 hour at room temperature. Images were acquired using ChemiDoc Touch System (Bio-Rad).

### NanoSight tracking analysis

Isolated exosomes were subjected to NanoSight tracking analysis (NTA) using a NanoSight LM10 instrument (NanoSight Ltd.). Settings were optimized and kept constant between samples. Each video was analyzed to give the mean, mode, median, and estimated concentration for each particle size. Data were processed using NTA 2.2 analytical software.

### OptiPrep linear gradient

Continuous density gradients were performed as described previously with adaptations. Briefly, 8 × 10^6^ cells cultured in a 10-cm culture dish were washed in cold PBS and resuspended in working solution [0.25 M sucrose, 4 mM MgCl_2_, 8.4 mM CaCl_2_, 10 mM EGTA, and 50 mM Hepes-NaOH (pH 7.0) with protease inhibitor cocktail]. Cells were lysed by sonication (three rounds of 3 s at 4°C) and centrifuged at 1000*g* for 5 min (2×). Continuous 5 to 30% OptiPrep gradients were prepared in working solution using a gradient mixer. Postnuclear supernatant (PNS) was added to the top of 9-ml gradients and centrifuged at 100,000*g* for 16 hours using a 70.1 Ti rotor. Sequential fractions were collected (1 ml each, from the top to the bottom of the tube). Fractions were recovered by ultracentrifugation at 100,000*g* for 30 min in an SW 32 Ti rotor. For WB, fractions were collected in PBS and further denaturated in Laemmli buffer.

### Isolation of EEs and LEs by sucrose density gradient centrifugation

For the separation of EEs and LEs, a protocol described by de Araujo *et al.* was used (*29*). ARPE-19 cells (25 × 10^6^), cultured in DMEM with 10% FBS, were washed with ice-cold PBS and collected with a cell scraper. Cells were collected to a 2-ml microfuge tube and centrifuged at 300*g* for 5 min at 4°C. Cell pellet was loosened using cold finger in a homogenization buffer [HB; 250 mM sucrose, 3 mM imidazole (pH 7.4), 1 mM EDTA, 0.03 mM cycloheximide, 10 mM iodoacetamide, 2 mM phenylmethylsulfonyl fluoride (PMSF), and 1× cocktail inhibitor from Sigma-Aldrich]. Samples were centrifuged at 1300*g* for 10 min at 4°C. Supernatant was discarded, and the pellet was gently resuspended with a wide-cut tip in three times the pellet volume of HB. The suspension was passed through a 25-gauge needle, attached to a 1-ml syringe, 10 times.

The homogenate was then diluted in HB (1 part homogenate to 0.7 parts HB) and centrifuged at 1600*g* for 10 min at 4°C. The supernatant was collected and centrifuged again. The PNS that originated from the second centrifugation is used for organelle isolation. PNS (5%) is centrifuged at 4°C for 10 min at 16,000*g*. The recovered supernatant was collected for cytoplasmatic fraction, free of vesicles. For the remaining PNS, the sucrose concentration was adjusted to 40.6%. The PNS was then loaded at the bottom of an ultracentrifuge tube. The 40.6% solution was overlaid with 1.5 volumes of 35% sucrose solution and 1 volume of 25% sucrose solution, and the tube was filled to the top with HB. The gradient was centrifuged at 210,000*g* for 16 hours at 4°C, using a 70.1 Ti rotor. Each interface was collected. LEs are found in the 25%/HB interface. EEs are present in the 35%/25% interface. The endosomal fractions were diluted in 35 ml of HB solution and centrifuged at 100,000*g* for 1 hour in an SW 32 Ti rotor. The organelle pellets were resuspended in an appropriate buffer depending on the downstream procedures.

For WB, fractions were collected in PBS and denaturated in Laemmli buffer. For in vitro uptake assays, fractions were collected in Mops buffer and protein concentration was determined. For endosome immunoprecipitation, endosomes were collected in KPBS (136 mM KCl, 10 mM KH_2_PO_4_, pH 7.25 was adjusted with KOH) buffer according to protocol (*56*).

### In vitro uptake of GST and GST-HIF1A assay

Recombinant proteins GST (SICGEN) and GST-HIF1A (Abnova, H00003091-P01) were incubated with 5 μg of freshly isolated vesicles and recombinant protein HSC70 (Enzo Life Sciences, ADI-SPP-751) in Mops buffer [10 mM Mops (pH 7.3), 0.3 M sucrose, 5.4 μM cysteine, and 1 mM DTT] and ATP regeneration buffer [1 mM ATP, 1 mM MgCl_2_, 7.5 mM phosphocreatine, and creatine kinase (50 μg/ml)] for 45 min at 37°C. After the incubation period, samples were cooled on ice for 1 min followed by a 30-min incubation with trypsin at 37°C. Samples were put back to ice and denaturated in Laemmli buffer, heated at 95°C for 5 min, and resolved on a 10% SDS–polyacrylamide gel electrophoresis (PAGE) gel. The gel was transferred to a nitrocellulose membrane for 75 min at 100 V. The membranes were blocked with TBS-T containing 5% skim milk, followed by incubation with antibodies against the proteins of interest.

### Rapid immunofluorescence for PAmCherry

Cells were grown on glass coverslips and fixed with 4% PFA (w/v) in PBS for 5 min at 37°C. Cells were then permeabilized with 0.05% saponin in PBS for 5 min at room temperature, washed with PBS, and incubated with 50 μl of primary antibody diluted in PBS containing 5% (v/v) FBS and 0.1% (w/v) bovine serum albumin (BSA) for 10 min at room temperature. The cells were then washed and incubated with secondary fluorochrome-conjugated antibodies diluted in PBS containing 5% (v/v) FBS and 0.1% (w/v) BSA for 10 min at room temperature. The cells were washed and viewed on the Zeiss LSM 980 Airyscan confocal microscope. For Rab5QL imaging, cells were transfected with a Rab5QL fused to GFP, and cells were incubated with antibody raised against one of the extracellular loops of CD63 for 30 min before fixation for immunofluorescence. The cells were washed and viewed on the Zeiss LSM 710 confocal microscope using a 63× 1.4 Plan-Apochromat oil-immersion objective. For 3D reconstruction of the images, the Imaris software was used.

### Live-cell imaging

Cells were cultured in μ-Slide eight-well chambered coverslips (Ibidi). As previously described (*17*), lysosomes were loaded with dextran 647 (0.5 mg/ml) in DMEM with 10% FBS for 4 hours at 37°C, followed by incubation in conjugate-free medium for 20 hours. For endosomes, the same cells were incubated with dextran 488 (0.5 mg/ml) in DMEM with 10% FBS for 10 min, followed by a chase of 10 min in conjugate-free medium. PAmCherry was photoactivated with a 405-nm laser for 1 min. Live cells were imaged in a Zeiss LSM 710 confocal microscope using a 63× 1.4 Plan-Apochromat oil-immersion objective. The images were acquired at 37°C under 5% CO_2_.

### Immunoprecipitation

Cells were collected from dishes with ice-cold PBS using a cell scraper and centrifuged at 15,000*g* for 10 min. In all cases, pellets (cells and exosomes) were resuspended in 150 μl of lysis buffer [50 mM tris-HCl (pH 7.4), 150 mM NaCl, 10 mM iodoacetamide, 2 mM PMSF, protease inhibitor cocktail, and 0.5% NP-40] and sonicated three times, 1 s each at 4°C. Afterward, samples were centrifuged at 15,000*g* for 10 min, and pellets were discarded. All samples were incubated with 2 μg of the antibody against the protein of interest overnight at 4°C. Subsequently, 30 μl of Protein G–Sepharose was added to the sample, and incubations proceeded at 4°C for 2 hours. Beads were washed three times with lysis buffer containing 0.15% NP-40, denatured with Laemmli buffer, and boiled at 95°C for 5 min. Samples were then analyzed by SDS-PAGE. The membranes were blocked with 5% nonfat milk in TBS-T and probed for the proteins of interest.

### Endosomal immunoprecipitation

Afterward, endosomal immunoprecipitation was adapted from protocol (*56*). Briefly, endosomes were gently resuspended in KPBS (136 mM KCl and 10 mM KH_2_PO_4_; pH 7.25 was adjusted with KOH). Magnetic beads were incubated with anti-LAMP2A or anti-LAMP2B antibodies for 1 hour. Subsequently, the endosomes were incubated with the magnetic beads for an additional hour at 4°C. Immunoprecipitates were then gently washed three times with KPBS. The samples were denatured with Laemmli buffer and boiled at 95°C for 5 min. Samples were then analyzed by SDS-PAGE. The membranes were blocked with 5% nonfat milk in TBS-T and probed for the proteins of interest.

### Subcellular fractioning

To evaluate the presence of exosomal HIF1A in the nuclei of receiving cells, exosomes were isolated from ARPE-19 cells that were under hypoxia with 300 μM CoCl_2_ for 12 hours. Subsequently, 5 × 10^5^ 769-P cells were incubated with 5 μg of exosomes for 1 hour. Cells were collected and fractioned. Briefly, cells were collected with a cell scraper with ice-cold PBS. Cells were centrifuged for 10 min at 300*g*. Cell pellet was resuspended in 50 μl of lysis buffer for 30 min on ice [50 mM tris-HCl (pH 7.4), 150 mM NaCl, 10 mM iodoacetamide, 2 mM PMSF, and 1× cocktail inhibitor from Sigma-Aldrich]. Samples were then centrifuged at 4°C for 10 min at 1000*g*. Supernatants were removed to a new 1.5-ml microfuge tube, and pellet (nuclear pellet) was lysed with Laemmli buffer. Supernatant was centrifuged at 4°C for 10 min at 16,000*g*. The resultant pellet (vesicular pellet) and the second supernatant (cytoplasm) were also lysed with Laemmli buffer. Samples were then analyzed by SDS-PAGE. The membranes were blocked with 5% nonfat milk in TBS-T and probed for the proteins of interest.

### Reporter assay

HIF1A activity was measured by transducing cells with the reporter gene Luciferase under the control of the HRE, using ONE-Glo Luciferase Assay System (Promega), according to the manufacturer’s specifications.

### Zebrafish in vivo experiments

Zebrafish lines were maintained in a circulating system with 14-hour day and 10-hour night cycle periods at 28°C. The mating and spawning of zebrafish were incited by the change of dark to light. Embryos were collected before they start independent feeding, at 5 days postfertilization (dpf). Therefore, no ethical approval was necessary according to the Council Directive 2010/63/EU on the protection of animals used for scientific purposes (*57*). Embryos were maintained at 28°C in E3 medium. For live imaging, *casper*, *Tg(kdrl:mCherry)*, or *Tg(mpeg1:mCherry)* embryos, at 100-cell stage, were injected in the YSL with pUbi-mCherry-p2A-GFP-ExoSignal in the presence or absence of MOs at 150 nM concentration, with a microinjector under a stereomicroscope. We used a Syntenin-a MO oligonucleotide directed against the translation start site [5′-ACAACGACATCCTTTCTGCTTTCA-3′ (*38*)], LAMP2A MO oligonucleotide directed against the LAMP2A unique intron-exon junction (5′-AGCTGAAAATAAAGAGAATGAGTGA-3′), and a standard control MO oligonucleotide (5′-CCTCCTACCTCAGTTACAATTTATA-3′). MOs were obtained from Gene Tools LLC (Philomath, OR). At 3 dpf, embryos were anesthetized in buffered MS-222 (200 μg/ml; Sigma-Aldrich) and incubated for 1 hour with 300 nM bafilomycin A before being immobilized in a 1% low melting agarose and imaged by Spinning Disk or Airyscan confocal microscopy (Zeiss LSM 980). For neovascularization assays at 3 dpf, 0.25 μg of exosomes was injected into the duct of Cuvier of *Tg(fli1a:EGFP)* zebrafish with a microinjector under a stereomicroscope. At 5 dpf, embryos were fixed with 4% PFA overnight at 4°C. Embryos were washed in PBS and mounted in glycerol mounting media. Images were acquired in a Zeiss LSM 710 confocal microscope using a 40× 1.2 C-Apochromat water-immersion objective. When required, optical slices were acquired, and 3D reconstruction of the images and quantifications were performed using Imaris software.

For exosome isolation, 20 to 40 zebrafish larvae were dissociated at 28°C using Liberase for 30 min with gentle agitation. Subsequently, exosomes were isolated from the samples as previously described.

### Statistical analysis

Data are reported as means ± SD of at least three independent experiments. Comparisons between multiple groups were performed by one-way analysis of variance (ANOVA) with Tukey’s multiple comparisons tests, using GraphPad Prism 8.0 software (GraphPad Software). For comparison between two groups, the paired *t* test was used. In all cases, *P* < 0.05 was considered significant. For in vivo experiments, sample size was determined, taking into account experimental feasibility and statistical significance. Animals and samples were randomized, and experimenters were blinded during experimental protocol and analysis.

## Notes

View/request a protocol for this paper from *Bio-protocol*.
