# Supplementary material for: LAMP2A regulates the loading of proteins into exosomes
Source: Sci Adv. 2022 Mar 25;8(12):eabm1140. doi: 10.1126/sciadv.abm1140 (PMC8956266; doi:10.1126/sciadv.abm1140)

Science Advances
AAAS

Supplementary Materials for

LAMP2A regulates the loading of proteins into exosomes

João Vasco Ferreira, Ana da Rosa Soares, José Ramalho, Catarina Máximo Carvalho, Maria Helena Cardoso, Petra Pintado, Ana Sofia Carvalho, Hans Christian Beck, Rune Matthiesen, Mónica Zuzarte, Henrique Girão, Guillaume van Niel, Paulo Pereira*

*Corresponding author. Email: paulo.pereira@nms.unl.pt

Published 25 March 2022, *Sci. Adv.* **8**, eabm1140 (2022)
DOI: 10.1126/sciadv.abm1140

The PDF file includes:

Figs. S1 to S8
Legends for movies S1 to S11
Legends for tables S1 to S3

Other Supplementary Material for this manuscript includes the following:

Movies S1 to S11
Tables S1 to S3

Supplementary Text

Supplemental Figure 1. Isolated sEVs are enriched in exosomal markers while LAMP2A KO does not interfere with endosomal morphology. A) ARPE-19 cells were transduced using lentiviral particles for the expression of spCas9 protein and the gRNA directed to the CT terminal of the LAMP2A isoform. Cells were grown in colonies until reaching around 50 cells per colony. Colonies were screened for the presence of LAMP2A by Western Blot. Western blot of whole cell lysates with antibodies against LAMP2, LAMP2A, LAMP2B and Actin (ACTB). LAMP2A KO does not affect the protein levels of the LAMP2B isoform. B) Cells were prepared according to the indicated protocols. Electron micrographs of WT and LAMP2A KO ARPE-19 cells show endosomes containing ILVs with the same morphology. C) ARPE-19 cells were incubated with Alexa 488 dextran to label endosomes and Magic Red dye to label lysosomes. We incubated cells for 15 min with the fluorescent Dextran, followed by a wash step, and took live cell images, using a confocal microscope, at specific time points to quantify the number of dextran vesicles that reached lysosomes. Live cell confocal microscopy images show colocalized and non-colocalized vesicles over 40 min. WT and LAMP2A KO cells show the same number of colocalized puncta in all time points. D) ARPE-19 cells were cultured in exosome-depleted medium. sEVs were isolated from cell culture supernatants from LAMP2A WT and KO (indicated in bold). The heatmap depicts intensity-based absolute quantification (iBAQ) based on MS across multiple samples. MS based analysis of sEVs show that the isolated vesicles are enriched in sEVs markers in a comparable level to three other studies (*19-21*). The proteins highlighted in red are cell organelle markers (CANX, endoplasmic reticulum; GOLGA2, golgi apparatus; NUP98, nuclear pore marker; BCL2, and outer mitochondrial membrane marker) which are depleted from the isolated sEVs fractions.

Supplemental Figure 2. mCherry-ExoSignal presence in sEVs. WT ARPE-19 cells were transduced with lentiviral particles for the expression of mCherry-Exosignal. sEVs were isolated from cell culture supernatants. A) Isolated sEVs were separated in a discontinuous sucrose gradient. Recovered fractions were blotted with antibodies raised against CD63 and mCherry. Results show that mCherry-Exosignal is present in vesicles from sucrose fractions that are enriched in the markers CD63 with densities that are typical of exosomes. B) Isolated sEVs were incubated with trypsin in the presence or absence of 1% of Triton X100. mCherry, unlike the membrane protein Cx43, is resistant to trypsin, indicating that mCherry-Exosignal is located in the inside of the sEVs. C) ARPE-19 cells expressing mCherry-Exosignal were transduced with adenoviral particles to express scramble shRNA or shRNA sequences for the depletion of Rab27a/b. The release of sEVs loaded with mCherry-ExoSignal is decreased by the depletion of Rab27. The results represent the mean ±SD of at least N=3 independent experiments (n.s. nonsignificant; $*p < 0.05$; $**p < 0.01$; $***p < 0.001$; $****p < 0.0001$).

Supplemental Figure 3. The PAmCherry-ExoSignal chimeric protein is sorted into early endocytic vesicles. Cells were transfected with Rab5QL-GFP and incubated for 30 min with antibody against CD63 extracellular loop for 30min before fixation. Immunofluorescence using confocal microscopy shows that mCherry-ExoSignal puncta localizes in Rab5QL-GFP

compartments, inside CD63 positive ILVs when LAMP2A is present. 3D images were reconstructed using Imaris software.

Supplemental Figure 4. Hypoxia Inducible Factor 1A (HIF1A) is present in sEVs and endosomal fractions. A) WT and LAMP2A KO ARPE-19 cells were cultured in exosome-depleted medium and incubated with 300 μM of the hypoxia-mimetic agent CoCl2 for 12h. Western Blot of cell extracts and exosomal fractions using antibodies raised against LAMP2A, HIF1A, FLOT1 and CD63 show that HIF1A is absent from LAMP2A KO exosomes. B) 769-P cells were transduced with lentiviral particles containing WTHIF1A and KFERQ-mutant HIF1A. Cells were cultured in exosome-depleted medium for 12h. Western Blot of cell extracts and exosomal fractions using antibodies raised against TSG101, Alix and HIF1A show that mutated HIF1A is not present in exosomes. C) WT and KO LAMP2A cell extracts were loaded on top of a continuous 5–30% optiprep gradient. Nine sequential fractions were collected. Western blot of isolate fractions with antibodies raised against endosomal markers CD63, FLOT1, LAMP2, LAMP2A, Alix, TSG101, Rab5 and Rab7 show HIF1A is present in endosomal fractions (7 and 8), only when LAMP2A is present. All samples were analyzed under the same experimental conditions. The results represent the mean ±SD of N=3 independent experiments (n.s. nonsignificant; *p < 0.05; **p < 0.01; ***p < 0.001; ****p < 0.0001).

Supplemental Figure 5. mCherry-ExoSignal colocalizes with early endosomal markers and e-MI substrates GAPDH and Aldolase are loaded into endosomes independently of LAMP2A. A) Immunofluorescence using LSM 980 Airyscan confocal microscopy of cells fixed in methanol with antibodies against mCherry, LAMP2A, EEA1 and m6p or Rab5-GFP. PAmCherry-ExoSignal shows high co-localization with LAMP2A, EEA1 and RAB5-GFP only when LAMP2A is present. B) Confocal microscopy of WT and LAMP2A Z-stack 3D reconstruction, using Imaris software, with antibodies raised against LAMP2A, LAMP2B and using Wheat Germ Agglutinin (WGA), conjugated to Alexa Fluor 488, as a membrane marker. LAMP2A and, to a lesser extent, LAMP2B are present at the plasma membrane. C) GAPDH-GST and Aldolase-GST was incubated with freshly isolated vesicles in the presence/absence of ATP, the molecular chaperone HSC70, and LAMP2A. Samples were treated with trypsin to degrade all the protein that was not protected by the endosomal membrane. Western blot using antibodies raised against the GST-tag show that both proteins are translocated into both EEs and LEs enriched fractions, independently of LAMP2A.

Supplemental Figure 6. mCherry-ExoSignal co-localizes with ESCRT-independent endosomal machinery. A) Confocal images of methanol fixated cells show recovery of colocalization between mCherry-ExoSignal and CD63, Alix or Rab31, but not with TSG101, VPS4b and Syn-1, when LAMP2A expression is rescued in LAMP2A KO cells. B) Quantification of KD efficiency. C) Immunofluorescence using confocal microscopy with antibodies raised against LAMP2A and LAMP2B. LAMP2A and LAMP2B do not completely co-localize.

Supplemental Figure 7. LAMP2A mediates loading of KFERQ-containing proteins into sEVs in the zebrafish larvae. A) Schematic representation of zebrafish experiment using mCherry-P2A-GFP-ExoSignal. B) *casper* zebrafish at 1000 cell stage were injected at the YSL with empty vector (sham) or GFP alone. Confocal microscopy using a spinning disk shows no GFP in the zebrafish larvae caudal plexus at 3dpf. C) WB of sEVs isolated from *casper* zebrafish

after injection with mCherry-P2A-GFP-ExoSignal. Only GFP-ExoSignal is present in isolated sEVs. D) sEVs were isolated from Zebrafish expressing human CD63-pHluorin injected at 1 cell stage in the presence or absence of Syntenin-a morpholino. Syntenin-a morpholino decreases sEVs levels. E) WB using antibodies raised against GFP and CD63 of extracts from larvae co-injected with the mCherry-P2A-GFP-ExoSignal construct and either CT, Syntenin-a or LAMP2A morpholino. Syntenin-a and LAMP2A morphilinos do not change GFP-ExoSignal and CD63-pHluorin levels. F) 3D reconstruction of *Tg(kdrl:mCherry)* larvae, expressing mCherry in endothelial cells, injected with sham or both GFP-ExoSignal and Syntenin-a morpholino. No GFP signal is visible in the caudal plexus. G) *Tg(mpeg1:mCherry)* larvae, expressing mCherry in macrophages, sham injected. No GFP signal is present in macrophages located in the caudal plexus. H) WT and KO LAMP2A ARPE-19 cells were cultured in exosome-depleted medium incubated with 300 μM of the hypoxia-mimetic agent $CoCl_2$ for 12h before exosome isolation from media supernatants. 769-P cells were incubated with the WT derived exosomes for 1h. Subsequently, cells were harvested and fractioned into Nuclear fraction (N), Cytoplasm fraction (C) and Vesicular fraction (V). Western Blot of the fractions using antibodies raised against HIF1A, Tubulin (TUB) and Lamin B, show that exosomal HIF1A is reaches the nucleus of the receiving cell. All samples were analyzed under the same experimental conditions. The results represent the mean ±SD of at least N=3 independent experiments (n.s. nonsignificant; $*p < 0.05$; $**p < 0.01$; $***p < 0.001$).

Supplemental Figure 8. Schematic representation of the proposed mechanism for cytosolic protein loading into exosomes. Cytosolic proteins containing KFERQ-like motifs are recognized by the chaperone HSC70 and targeted to the endosomal membrane, where it binds to the receptor LAMP2A. As the endosomal membrane invaginates to create ILVs, proteins are trapped in their lumen while binding to LAMP2A. The LAMP2A-mediated loading of proteins is assisted by additional molecular machinery, including CD63, Alix, Syntenin-1, Rab31 and ceramides, rather than ESCRT molecular components such as TSG101 and VPS4b. Subsequently, endosomes filled with ILVs travel to the cell periphery, in a process likely mediated by Rab27, fusing with the plasma membrane and releasing its contents as exosomes.

Supplemental movie 1. GFP-ExoSignal is present in the circulation network of the caudal plexus in zebrafish. Sample 1 movie of the zebrafish shows the caudal artery, the caudal vein and a complex venous vascular network. *casper* zebrafish at 1000 cell stage were co-injected at the YSL with the GFP-ExoSignal-P2A-mCherry construct with Control Morpholino. Movie shows that GFP-ExoSignal is present in the caudal plexus at 3dpf.

Supplemental movie 2. GFP-ExoSignal is present in the circulation network of the caudal plexus in zebrafish. Sample 2 movie of the zebrafish shows the caudal artery, the caudal vein and a complex venous vascular network. *casper* zebrafish at 1000 cell stage were co-injected at the YSL with the GFP-ExoSignal-P2A-mCherry construct with Control Morpholino. Movie shows that GFP-ExoSignal is present in the caudal plexus at 3dpf.

Supplemental movie 3. GFP-ExoSignal is present in the circulation network of the caudal plexus in zebrafish. Sample 3 movie of the zebrafish shows the caudal artery, the caudal vein and a complex venous vascular network. *casper* zebrafish at 1000 cell stage were co-injected at the

YSL with the GFP-ExoSignal-P2A-mCherry construct with Control Morpholino. Movie shows that GFP-ExoSignal is present in the caudal plexus at 3dpf.

Supplemental movie 4. GFP signal is absent from the circulation network of the caudal plexus of zebrafish in sham condition. Movie of the zebrafish shows the caudal artery, the caudal vein and a complex venous vascular network. *casper* zebrafish at 1000 cell stage were injected at the YSL with vehicle showing no GFP signal at 3dpf.

Supplemental movie 5. Untagged GFP is absent from the circulation network of the caudal plexus in zebrafish. Movie of the zebrafish shows the caudal artery, the caudal vein and a complex venous vascular network. *casper* zebrafish at 1000 cell stage were injected at the YSL with a GFP construct. Movie shows that untagged GFP is absent from the circulation of the caudal plexus at 3dpf.

Supplemental movie 6. GFP-ExoSignal particles are present in the circulation network of the caudal plexus in zebrafish. Movie showing GFP-ExoSignal sEVs circulating in the caudal plexus.

Supplemental movie 7. GFP-ExoSignal particles are present in the circulation network of the caudal plexus of zebrafish. Movie showing GFP-ExoSignal sEVs circulating in the caudal plexus and attaching to the vessel wall.

Supplemental movie 8. Syntenin-a morpholino inhibits GFP-ExoSignal presence in the circulation network of the caudal plexus in zebrafish. Movie of the zebrafish shows the caudal artery, the caudal vein and a complex venous vascular network. *casper* zebrafish at 1000 cell stage were co-injected at the YSL with the GFP-ExoSignal-P2A-mCherry construct and with Syntenin-a Morpholino. Movie shows that Syntenin-a morpholino inhibits GFP-ExoSignal presence from the caudal plexus at 3dpf.

Supplemental movie 9. LAMP2A morpholino inhibits GFP-ExoSignal presence in the circulation network of the caudal plexus of zebrafish. Movie of the zebrafish shows the caudal artery, the caudal vein and a complex venous vascular network. *casper* zebrafish at 1000 cell stage were co-injected at the YSL with the GFP-ExoSignal-P2A-mCherry construct and with LAMP2A Morpholino. Movie shows that LAMP2A morpholino inhibits GFP-ExoSignal presence from the caudal plexus at 3dpf.

Supplemental movie 10. GFP-ExoSignal is present in macrophages at the caudal plexus of zebrafish. Movie of zebrafish caudal plexus. *Tg(mpeg1:mCherry)* larvae, expressing mCherry in macrophages, were injected at the YSL at 1000 cell stage with the GFP-ExoSignal construct. Movie shows that GFP-ExoSignal is present in macrophages located in the caudal plexus.

Supplemental movie 11. Untagged GFP is absent from macrophages at the caudal plexus of zebrafish. Movie of zebrafish caudal plexus. *Tg(mpeg1:mCherry)* larvae, expressing mCherry in macrophages were injected at the YSL at 1000 cell stage with the untagged GFP construct. Movie shows that GFP alone is absent from macrophages located in the caudal plexus.

Supplemental Table S1. List of proteins identified in LC-MS/MS analysis of sEVs fractions.

Supplemental Table S2. List of proteins containing a KFERQ motif identified in LC-MS/MS analysis of sEVs fractions.

Supplemental Table S3. List of downregulated proteins identified in LC-MS/MS analysis of sEVs fractions.

Supplemental Figure 1

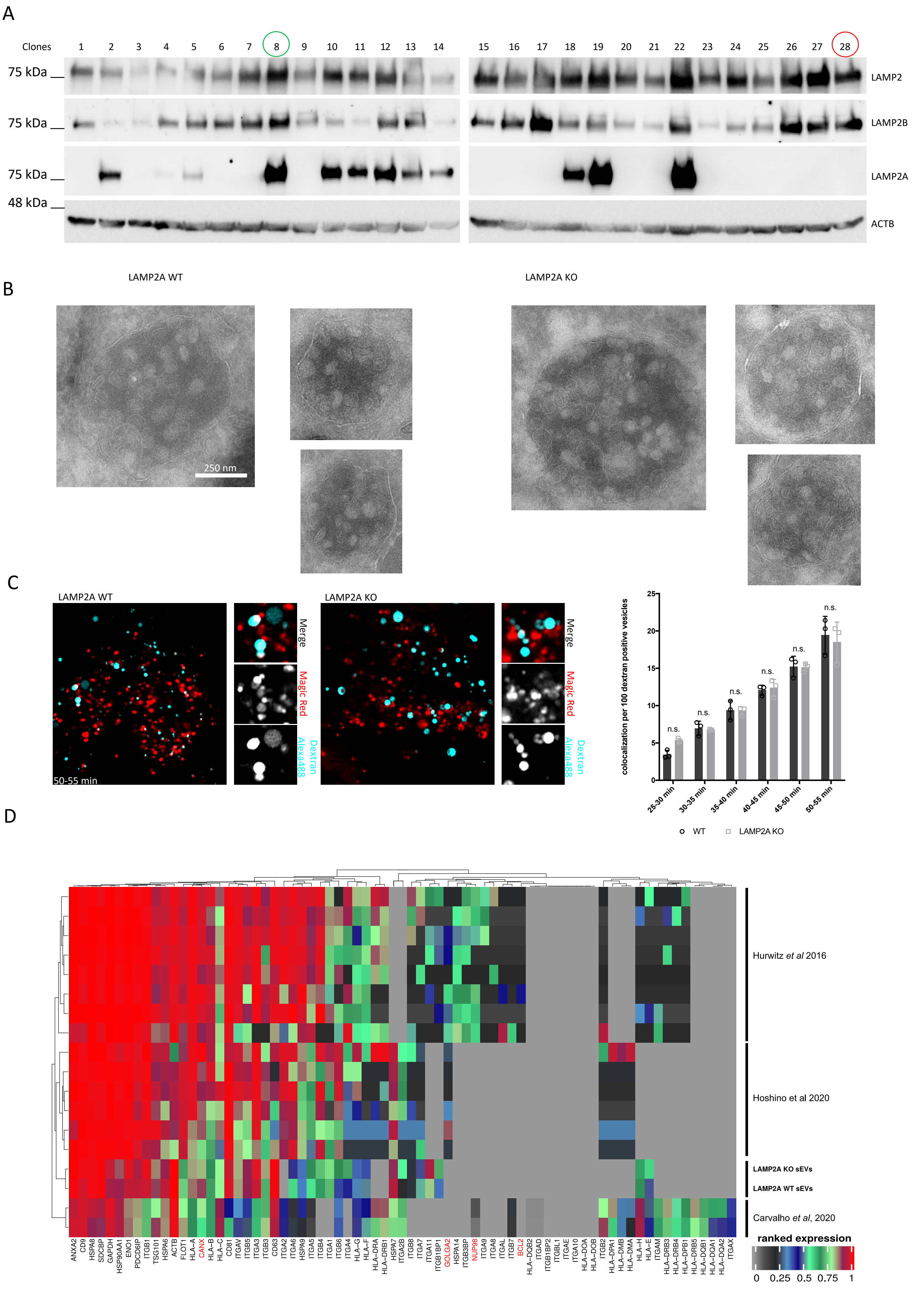

Supplemental Figure 2

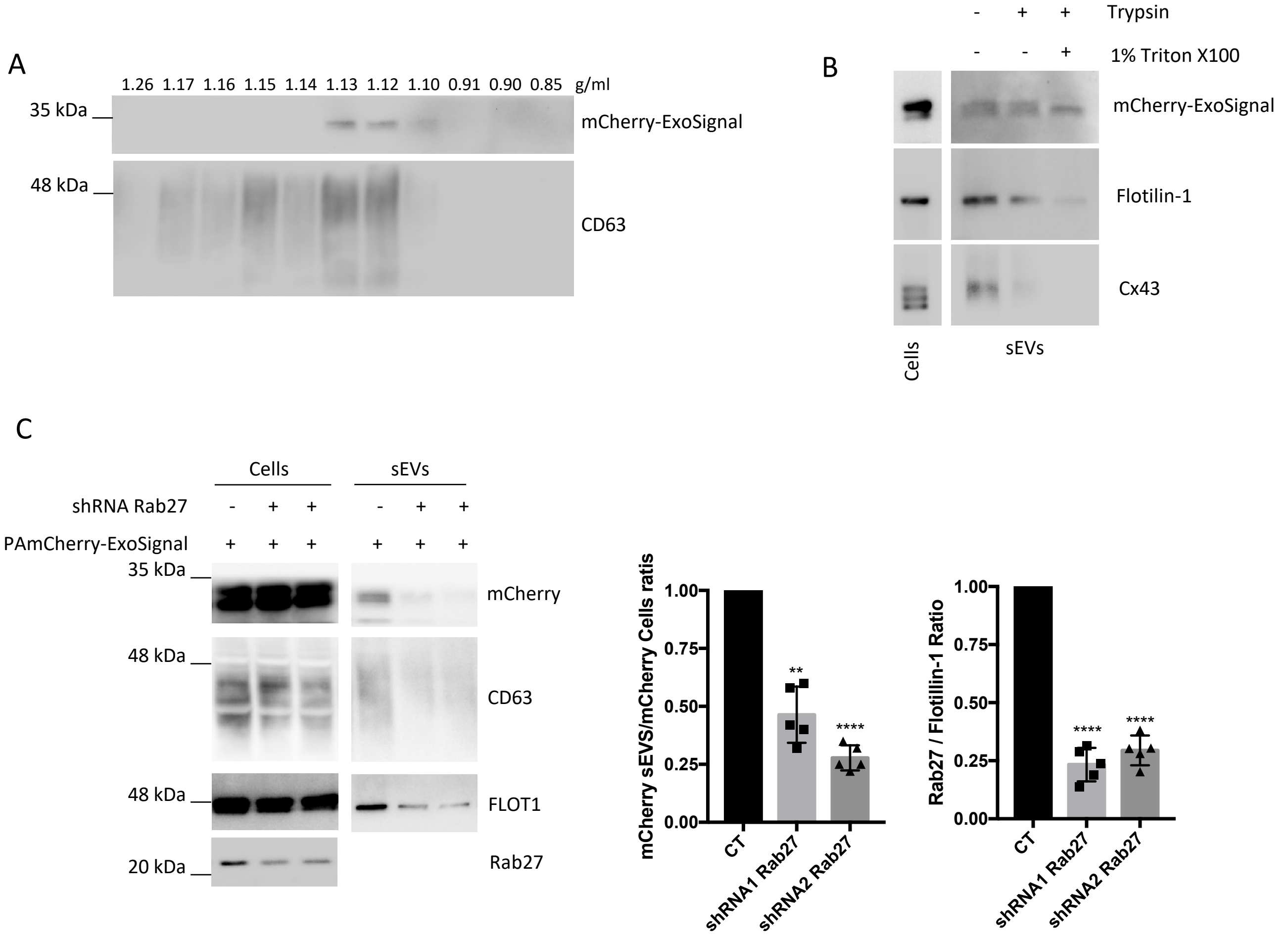

Supplemental Figure 3

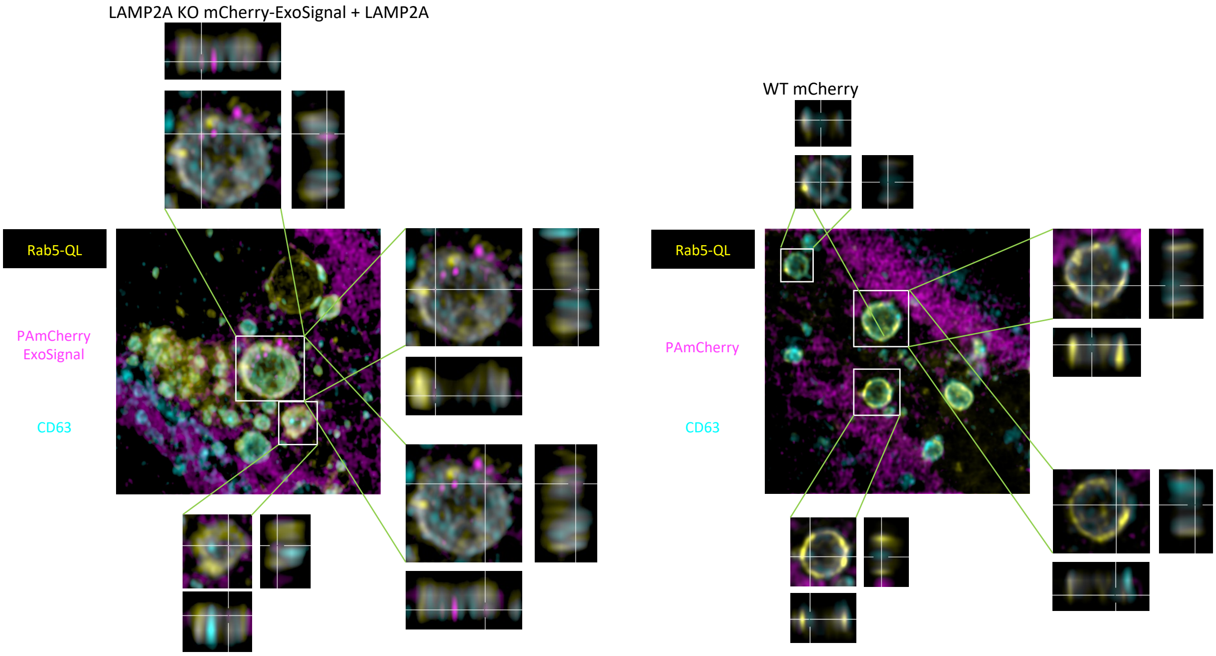

Supplemental Figure 4

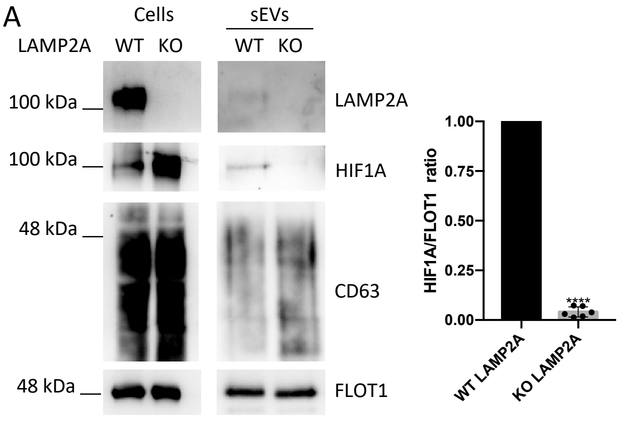

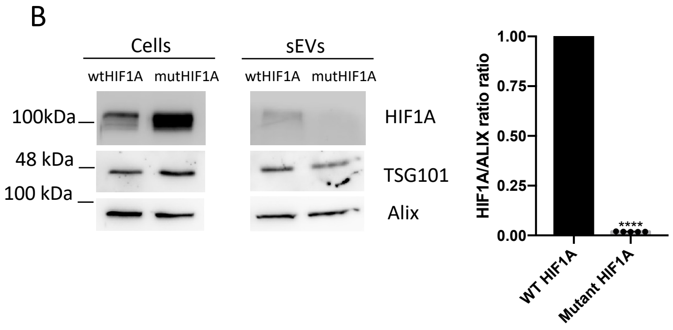

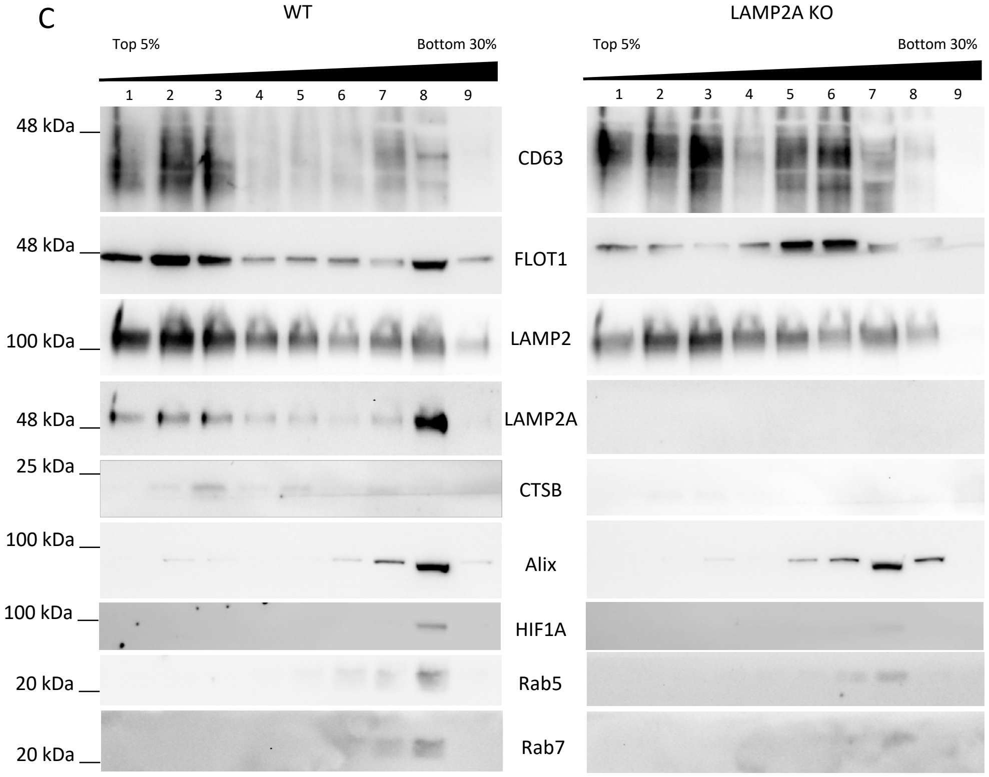

Supplemental Figure 5

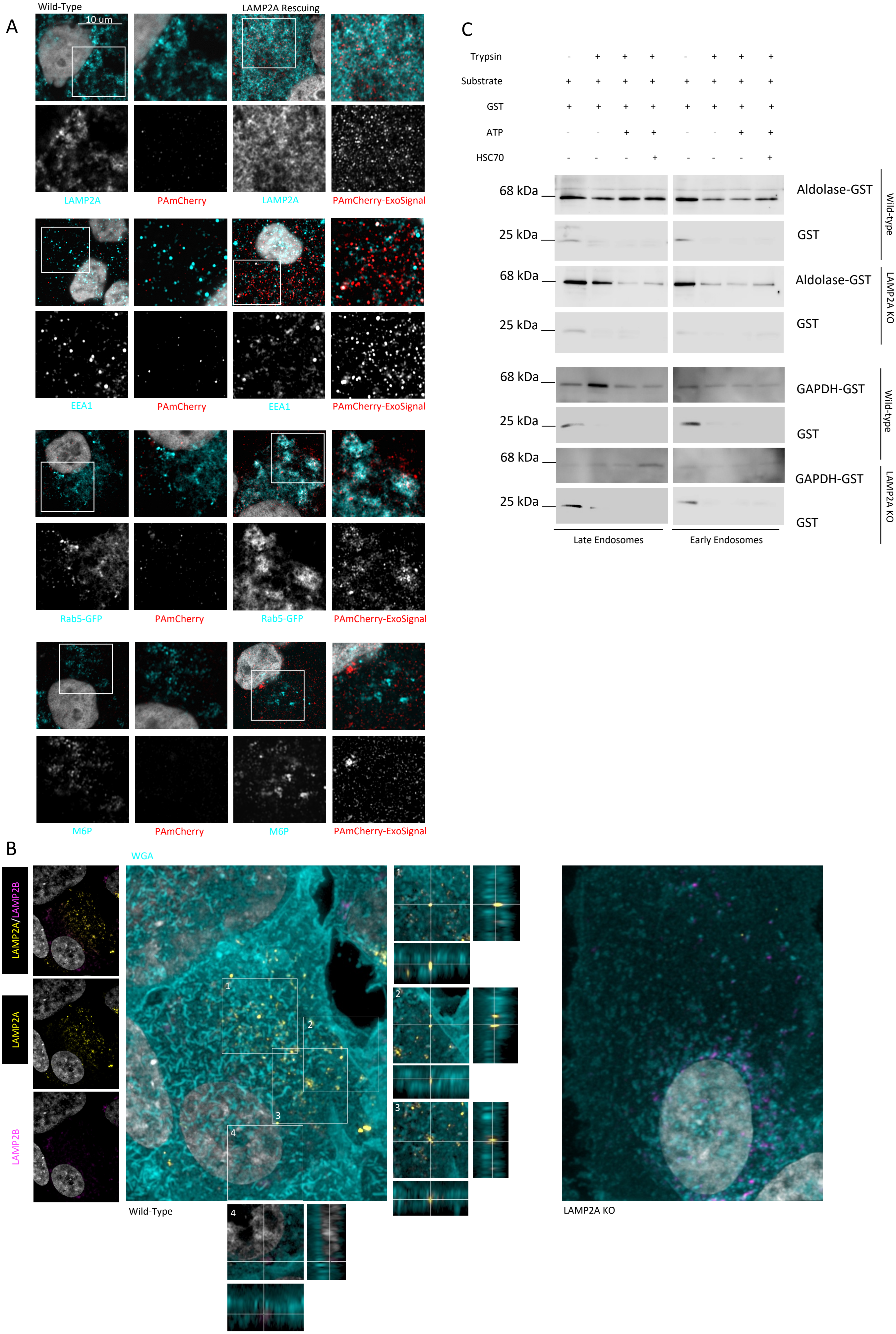

Supplemental Figure 6

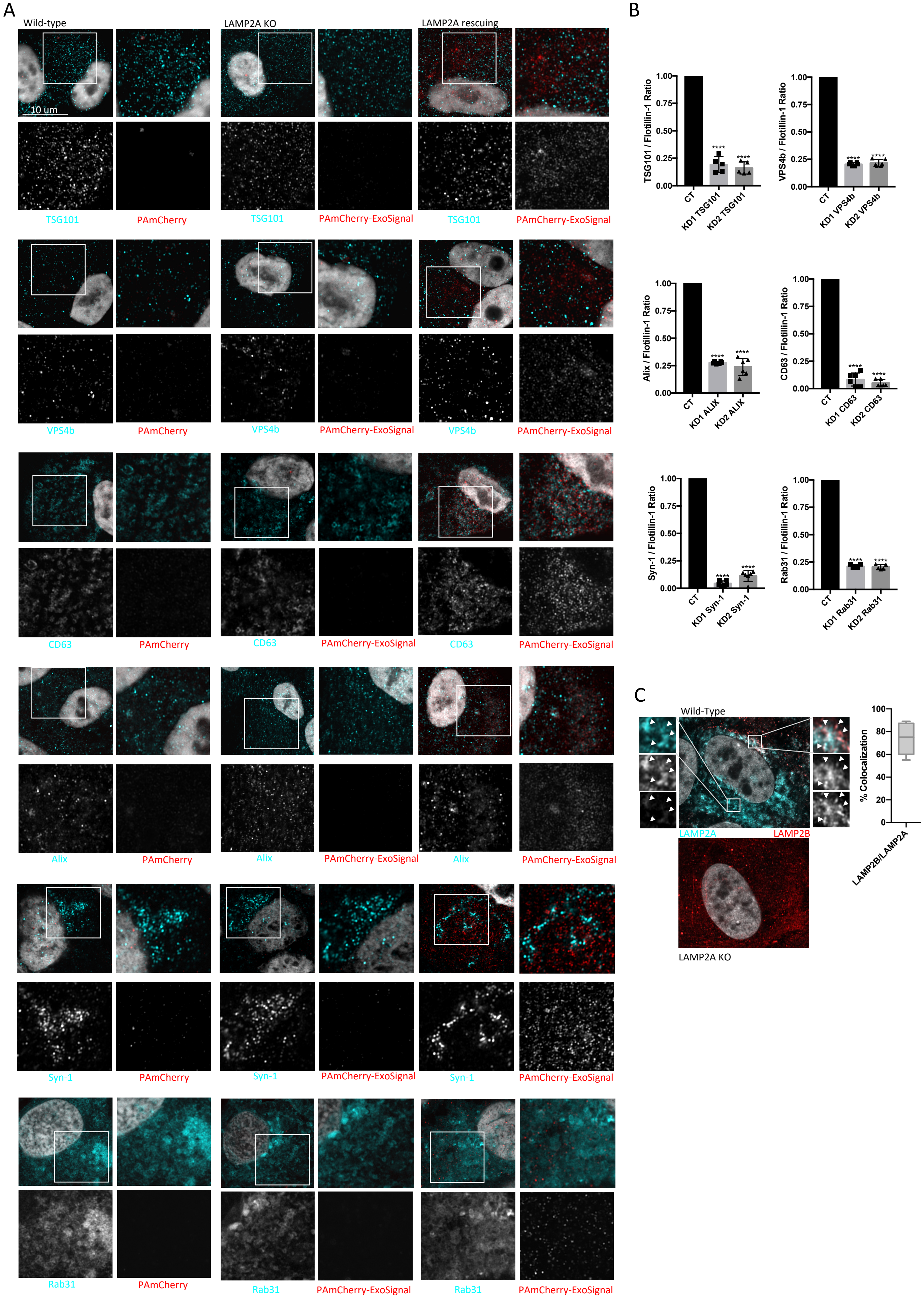

Supplemental Figure 7

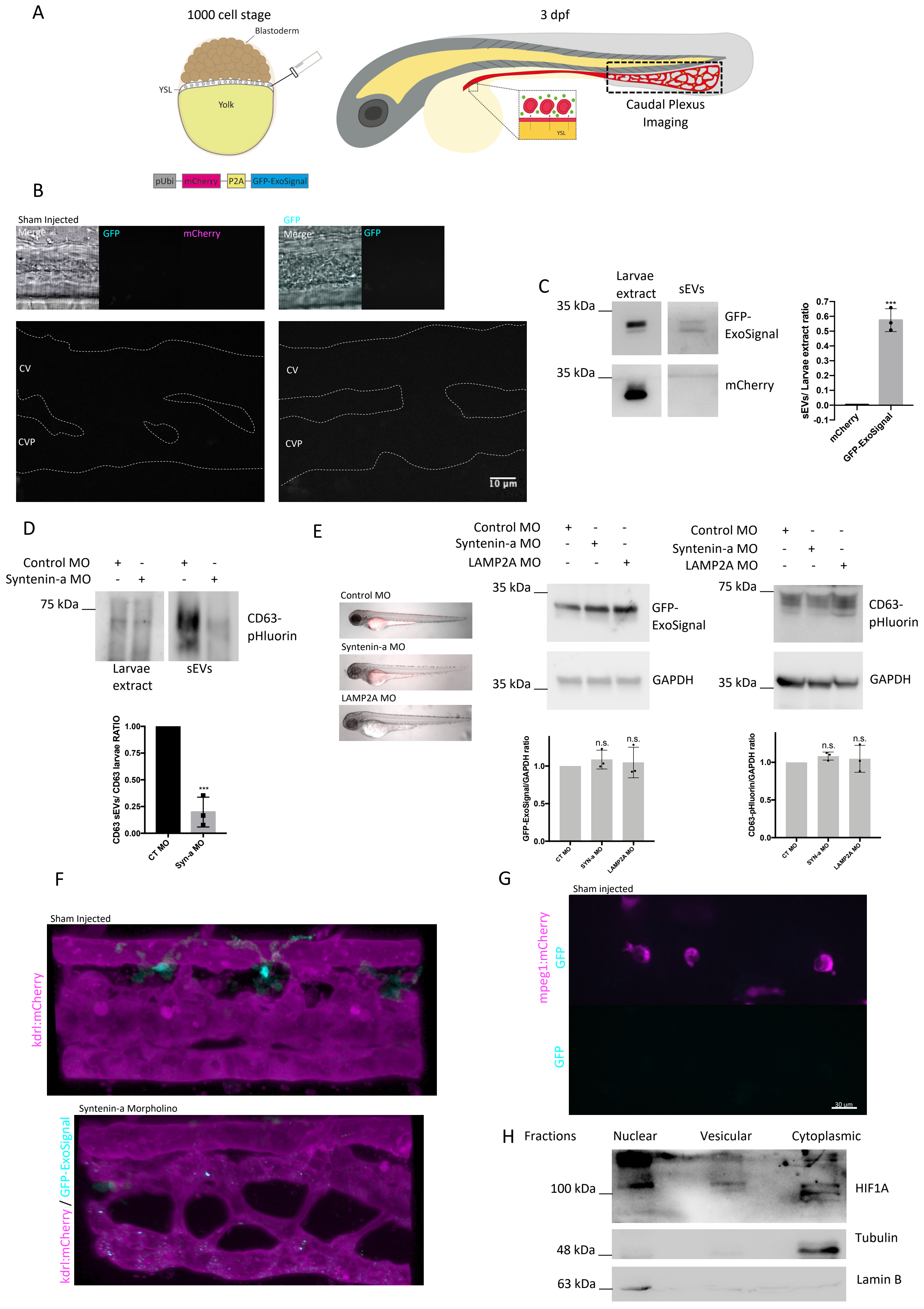

Supplemental Figure 8

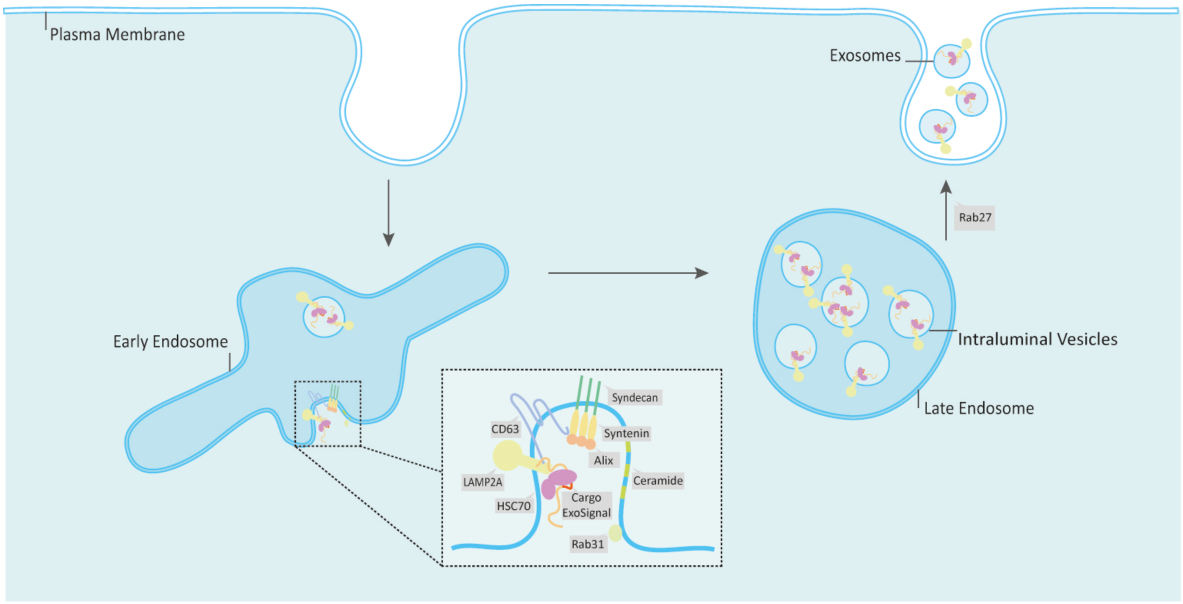

Supplement: Supplementary file 1 — Figs. S1 to S8 [file sciadv.abm1140_sm.pdf]
